# Inhibition of adult hippocampal neurogenesis induced by postoperative CD8 + T-cell infiltration is associated with cognitive decline later following surgery in adult mice

**DOI:** 10.1186/s12974-023-02910-x

**Published:** 2023-10-05

**Authors:** Xiaowei Li, Hong Wang, Qidi Zhang, Xiaobin Sun, Mengyuan Zhang, Gongming Wang

**Affiliations:** 1 Department of Anesthesiology, Shandong Provincial Hospital, Shandong University, Jinan, 250021 Shandong China; 2 Department of Anesthesiology, Shandong Provincial Hospital affiliated to Shandong First Medical University, Jinan, 250021 Shandong China; 3 Department of Nephrology, Tai’ an Central Hospital, Taian, 271000 Shandong China

**Keywords:** Postoperative cognitive decline, Adult hippocampal neurogenesis, T-cell infiltration, IFN-γ, IFNGR1

## Abstract

**Background:**

Some patients show persistent cognitive decline for weeks, months or even years after surgery, which seriously affects their long-term prognosis and quality of life. However, most previous basic studies have focused mainly on the mechanisms of early postoperative cognitive decline, whereas cognitive decline in the longer term after surgery is less well-understood. The subgranular zone of the dentate gyrus exhibits life-long neurogenesis, supporting hippocampus-dependent learning and memory.

**Main text:**

The aim of this study was to investigate whether adult hippocampal neurogenesis (AHN) involves in cognitive decline later following surgery and to further explore the roles of CD8 + T lymphocytes infiltrating the hippocampal parenchyma after surgery in this pathological process. Cognitive function was examined in adult mice that underwent laparotomy combined with partial hepatectomy, and the results showed that cognitive decline persisted in mice who underwent surgery during the first postoperative month, even though there was a trend toward continuous improvement over time. Significantly decreased numbers of DCX + cells, BrdU + cells, and BrdU + /DCX + cells were observed on day 8 after surgery, and a significantly decreased number of NeuN + /BrdU + cells was observed on day 28 after surgery, which indicated inhibition of AHN. After surgery, T lymphocytes, the majority of which were CD8 + T cells, infiltrated the hippocampus and secreted Interferon-γ (IFN-γ). Depletion of CD8 + T cells could inhibit the increase of IFN-γ synthesis, improve hippocampal neurogenesis, and improve postoperative cognitive function. Hippocampal microinjection of IFN-γ neutralizing antibody or adeno-associated virus to knock down IFN-γ receptor 1 (IFNGR1) could also partially attenuate the inhibition of AHN and improve postoperative cognitive function.

**Conclusions:**

These results demonstrate that postoperative infiltration of CD8 + T cells into the hippocampus and subsequent secretion of IFN-γ contribute to the inhibition of AHN and cognitive decline later following surgery.

**Supplementary Information:**

The online version contains supplementary material available at 10.1186/s12974-023-02910-x.

## Introduction

Cognitive decline is a common neurological complication following anesthesia and surgery that impairs higher cerebral cortical functions, such as memory, concentration, and information processing. A multispecialty group of experts from around the world revised the nomenclature and classification of cognitive change associated with anesthesia and surgery in 2018 [1]. The new classification includes cognitive decline diagnosed before operation, any form of acute event within 1 week after surgery or before discharge (postoperative delirium, POD), cognitive decline diagnosed up to 30 days after the procedure (delayed neurocognitive recovery, DNR) and decline up to 12 months (postoperative neurocognitive disorder, pNCD). Different underlying neuropathogenesis may underlie the generation of different categories of postoperative cognitive decline [2]. Most previous basic studies have focused mainly on the mechanisms of early postoperative neurocognitive decline, whereas cognitive decline later following surgery is less well-understood. Although postoperative cognitive decline improves over time, the incidences of DNR and pNCD are not low. Reportedly, neurocognitive dysfunction is present in 36.6% of young, 30.4% of middle-aged, and 41.4% of elderly patients at hospital discharge [3]. There have also been reports that among patients older than 55 years undergoing major elective noncardiac surgery, cognitive decline occurs in 54.3% of patients at 6 weeks and 46.1% at 1 year [4]. DNR and pNCD not only increase the costs and family burden but also impact short- and long-term outcomes [5]. For example, research has shown that patients with DNR or pNCD are at an increased risk of death in the first year after surgery [3].

Although cognitive function engages multiple brain regions, postoperative cognitive decline appears to be restricted to hippocampus-dependent cognition [6, 7]. For adult mammalian or rodent brains, neural stem cells in the subgranular zone (SGZ) of the hippocampal dentate gyrus retain the potential for self-renewal and continuously generate new neurons to incorporate into the granule cell layer of the dentate gyrus [8, 9], which play an important role in hippocampus-dependent cognition. Numerous studies have reported that defects in neurogenesis contribute to several human neurological and psychiatric diseases, such as Alzheimer’s disease and depressive-like behavior [10, 11], while the promotion of neurogenesis improves cognitive function [12, 13]. However, few studies have explored the association between adult hippocampal neurogenesis (AHN) and cognitive decline later following surgery.

Previous studies have revealed that neuroinflammation, and particularly the innate immune system represented by microglia, is a major contributor to the development of postoperative cognitive decline [14]. However, until now, little has been known about the contribution of adaptive immune responses to the pathophysiology of postoperative cognitive decline. With increasing age, the permeability of the blood‒brain barrier increases, and the levels of leukocyte chemokines in the brain parenchyma increase, which contribute to lymphocyte infiltration into the brain [15, 16]. T-cell infiltration, primarily CD8 + T cells, has been found in aging process and many neurodegenerative diseases, and can potentiate inflammation, inhibit neurogenesis and impair cognition [16–19]. Reversible disruption of the blood‒brain barrier has been confirmed to occur in several animal models of postoperative cognitive decline [20, 21], which facilitates T-cell infiltration into the brain parenchyma. Our previous research also found that the hippocampus was highly likely to be infiltrated with leukocytes after surgery (Additional file 1: Figure S1). Interferon-γ (IFN-γ) is a pleiotropic cytokine that is produced mainly by T lymphocytes and natural killer cells. A clinical study has shown that the levels of IFN-γ in cerebrospinal fluid are significantly increased after orthopedic surgery [22]. IFN-γ receptor (IFNGR) can be detected on the surfaces of neural stem cells and immature neurons. Both in vitro and in vivo experiments have indicated that IFN-γ can inhibit neural stem cell proliferation and reduce neurogenesis [17, 23]. High expression of IFNGR has also been observed on the surface of microglia which has been validated to play an important role in postoperative cognitive decline [17, 24]. However, the effects of T cells and IFN-γ on postoperative AHN and cognitive decline later following surgery have not been reported.

In this study, we subjected adult mice to laparotomy combined with partial hepatectomy and examined their cognitive function at weeks 2, 3 or 4 after surgery. We observed inhibition of AHN after surgery by double immunofluorescence staining, examined T-cell infiltration in the hippocampus after surgery and further classified T cells. The roles of T cells in postoperative AHN inhibition and cognitive decline at week 4 after surgery were verified by administering an anti-CD8 monoclonal antibody. We further explored the roles of IFN-γ secreted by infiltrated CD8 + T cells in the inhibition of AHN and postoperative cognitive impairment by administering an IFN-γ neutralizing antibody or knocking down IFNGR1. We demonstrate that postoperative infiltration of CD8 + T cells into the hippocampus and subsequent secretion of IFN-γ can contribute to the inhibition of AHN and cognitive decline later following surgery.

## Materials and methods

### Animals

A total of 388 twelve-month-old male C57BL/6 mice (Beijing Vital River Laboratory Animal Technology Co., Ltd.) were used for the experiments. Research has shown that 10–14-month-old mice correspond to 38–47-year-old humans [25, 26]. A 12-month-old mouse is approximately equivalent to a 43.7-year-old adult human [27]. Although not classified as elderly mice, 12-month-old mice have obtained some biomarkers of aging [25]. To avoid the effects of BrdU injection on the conclusions of this study, the results of behavioral tests and immunofluorescence staining were from independent sets of mice. Hippocampal tissues (for PCR or ELISA) were taken immediately after blood samples (for ELISA or measuring hemoglobin levels) were collected. Therefore, the numbers in Fig. 3C–E and Table 1 overlapped, and the numbers in Fig. 4C, and D overlapped. Animals were housed three per cage under standard illumination parameters (12-h light/dark cycle) and had ad libitum access to laboratory chow and water.

**Table 1:** Blood analysis of mice

	Control	Surgery	*p* value
PaO_2_ (mmHg)	90.8 ± 1.1	90.0 ± 1.6	0.411
Lactic acid (mmol/L)	1.1 ± 0.1	1.2 ± 0.2	0.468
Hb (g/dL)	13.6 ± 0.7	14 ± 0.3	0.372

*N* = 4/group

### Surgical procedure

The animals were anesthetized by intraperitoneal injection of 1.25% tribromoethanol (0.2 ml/10 g). A 2-cm midline incision was made on the upper abdomen. An index finger covered in a sterile glove was inserted into the peritoneal cavity, and the viscera and musculature were vigorously manipulated. A segment of approximately 10 cm of the small intestines was then exteriorized. After vigorous rubbing between the thumb and index finger for 30 s, the intestines were returned to the peritoneal cavity. The liver was then isolated, and the common hepatic pedicle of the left lateral lobe and the middle lobe was ligated tightly. Once ischemia presented, the corresponding liver lobes were removed carefully. The peritoneum and skin were sutured with silk. The whole procedure was strictly aseptic. Body temperature was maintained by a heat lamp during surgery. Buprenorphine (0.1 mg/kg) was injected subcutaneously after the operation for analgesia. The mice in the control group were only given the same dose of general anesthesia and postoperative analgesia. None of the experimental animals died due to anesthetic/surgical complications. Oxygen partial pressure and lactic acid were monitored immediately after surgery or anesthesia by percutaneous cardiac puncture. Mice that underwent cardiac puncture were euthanized. Hemoglobin levels were monitored 1 day after surgery or anesthesia by retro-orbital puncture.

### Behavior tests

#### Morris water maze (MWM) test

**Fig. 1:**
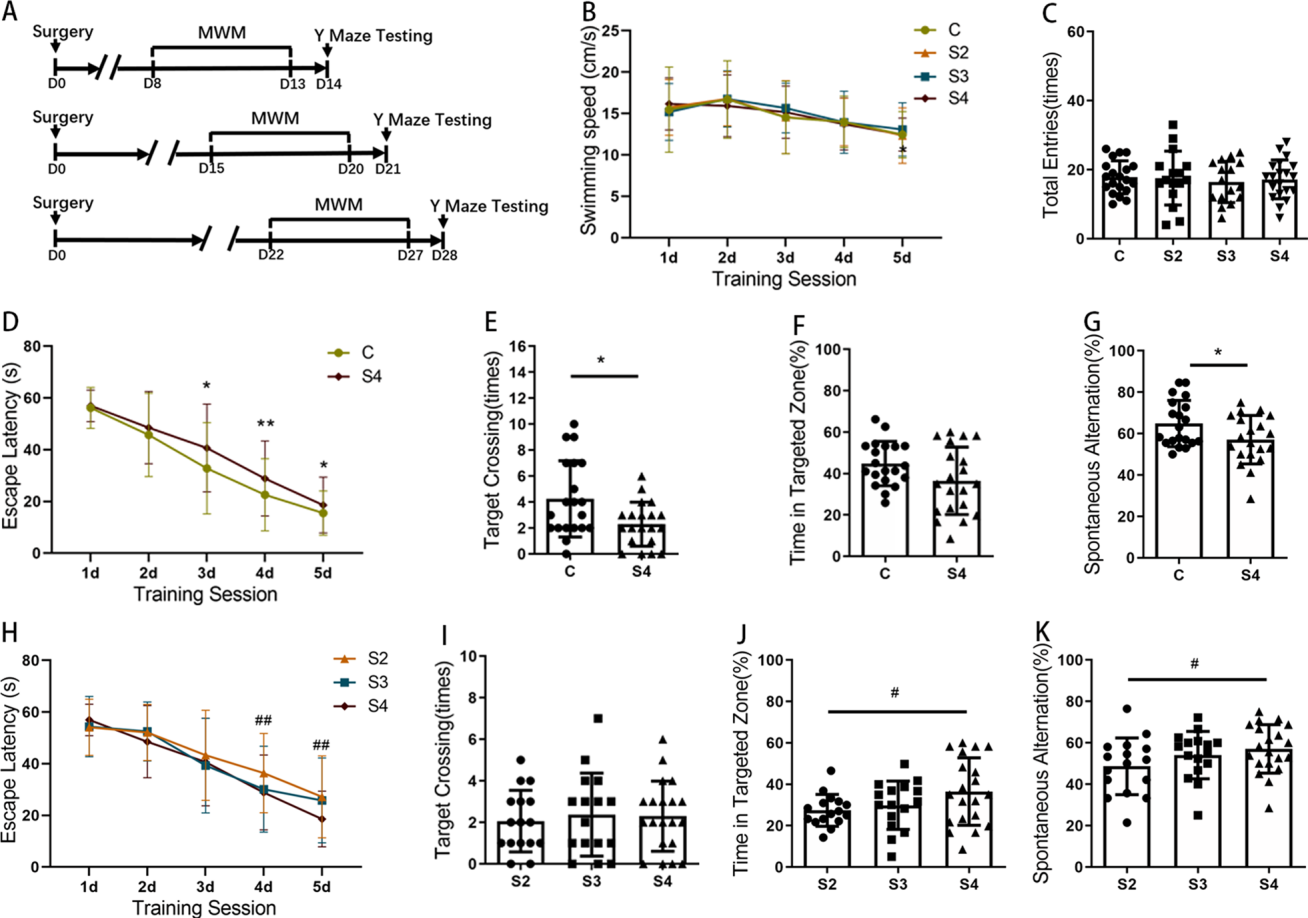
Postoperative cognitive decline persists up to the fourth week following surgery in adult mice. **A** Experimental protocol diagram; **B** swimming speeds of mice during training days in the MWM; **C** the number of total arm entries in the Y maze; **D**, **H** Escape latencies during training days in the MWM; **E**, **I** Times of target crossings on the testing day in the MWM; **F**, **J** Percentages of time spent in the target zone on the testing day in the MWM; **G**, **K** Spontaneous alternation rate in the Y maze. D, day post operation; MWM, Morris water maze; C, control; S2/S3/S4, animals that underwent behavioral testing at weeks 2/3/4 after surgery. *N* = 16–20/group. S4 vs. C, *p < 0.05, **p < 0.01; S4 vs. S2, ^#^*p* < 0.05, ^##^*p* < 0.01

The water maze was an opaque circular pool with a diameter of 150 cm and a height of 60 cm. The water temperature was maintained at 25 ± 2 °C. The maze was divided into 4 quadrants by specific marks on each quadrant boundary. In the target quadrant, an invisible platform with a diameter of 10 cm was placed 1 cm below the water surface. The water maze protocol started on days 8, 15 or 22 after surgery (Fig. 1a). For hidden platform training, mice were given four training trials daily for five consecutive days. Each mouse was released into the water from one of the four quadrant boundaries facing the wall of the test pool, and the order of the quadrants was chosen at random. The time for the mouse to find the platform and stay on the platform for more than 2 s was recorded as the escape latency time. Any mouse that failed to find the platform within 60 s was guided to the platform and allowed to stay there for 15 s, and then the time was recorded as 60 s. The platform was removed from the pool on the sixth day for the probe test. Each mouse was released into the water from the opposite side of the target quadrant and was allowed to explore the maze for 60 s. The number of times for the mouse crossing the previous exact location of the platform and the percentage of time spent in the target quadrant were recorded using tracking software. Swimming speed was calculated to exclude the possibility that any learning or memory deficits observed in the MWM were due to deficits in motor activity. After every trial, the mouse was allowed to recover on a warm blanket for at least 1–2 min before being returned to its regular cage.

#### Y-maze spontaneous alternation test

Each mouse was placed in a random arm of the symmetrical Y-maze and was free to explore the maze for 5 min to assess spontaneous alternating behavior. A correct alternation was defined as entry into three different arms consecutively. The spontaneous alternation response rate was calculated as follows: spontaneous alternation rate = the number of correct alternations/(the number of total entries into the arms − 2) × 100%. At the end of each test, the maze was wiped with 75% alcohol.

### Bromodeoxyuridine injections

Bromodeoxyuridine (BrdU, Sigma, USA) was dissolved in normal saline (10 mg/ml). After surgery, the animals were given two daily injections of 100 mg/kg i.p. BrdU at an interval of more than 8 h for 5 consecutive days.

### Immunofluorescence

Animals were transcardially perfused with normal saline followed by 4% paraformaldehyde. The whole brain was removed and placed in 4% paraformaldehyde for 24–48 h before being dehydrated in 30% sucrose overnight at 4 °C. The brain was embedded in optimum cutting temperature compound and cut into 9-μm-thick coronal sections on a freezing microtome. Antigen retrieval was achieved by heating in Tris–EDTA buffer using a pressure cooker. For BrdU staining, sections were incubated in 2 M HCl at 37 °C for 10 min. Then, the sections were blocked for 30 min with 10% normal donkey serum containing 0.1% Triton X-100 and incubated with primary antibodies (rabbit anti-Doublecortin, 1:200, Abcam; mouse anti-BrdU, 1:200, CST; rabbit anti-NeuN, 1:200, Abcam; rabbit anti-CD3, 1:800, Abcam) in 10% normal donkey serum at 4 °C overnight. On the following day, the sections were incubated with secondary antibodies (goat anti-rabbit IgG, 1:1000, Abcam; goat anti-mouse IgG, 1:1000, Abcam) for 50 min at room temperature. DAPI was used for counterstaining. The sections were immediately observed under a fluorescence microscope (OLYMPUS BX53M) and photographed.

### Flow cytometry

Animals were transcardially perfused with precooled PBS. Then, the whole brain was removed and placed in precooled complete DMEM to further isolate the hippocampus under a stereomicroscope. The hippocampi were scissor-minced and manually homogenized. The tissue was subsequently dissociated by adding accutase (Sigma-Aldrich) and incubated at 37 °C, 5% CO_2_ in a cell culture incubator for 15 min. The reaction was stopped by adding serum-containing culture medium. Single-cell suspensions were filtered with a 70-µm nylon mesh. Density gradient centrifugation using Percoll (Biosharp) was performed to isolate the mononucleated immune cells. Samples were washed with PBS three times. Fc receptors were blocked by TruStain FcX™ PLUS (anti-mouse CD16/32, Biolegend) for 5 min on ice. PE anti-mouse CD3ε antibody (Biolegend), FITC anti-mouse CD4 antibody (Biolegend) and APC anti-mouse CD8a antibody (Biolegend) were added for 20 min at 4 °C and protected from light. Isotype-matched antibodies were used as controls. After cleaning the cells with PBS three times, samples were run on a BD LSRFortessa cell analyzer.

### Quantitative PCR

The TRIzol reagent method was used to extract RNA from the hippocampus. The RNA concentration was measured using an ultramicro-spectrophotometer (NanoDrop 2000). cDNA was obtained using Evo M-MLV RT Premix for qPCR (Accurate Biotechnology, China). Real-time PCR was performed using a SYBR Green Premix Pro Taq HS qPCR Kit (Accurate Biotechnology, China) under the following conditions: 30 s at 95 °C, 40 cycles at 95 °C for 5 s and 60 °C for 30 s. The primer sequences were as follows: β-actin forward: CATCCGTAAAGACCTCTATGCCAAC; β-actin reverse: ATGGAGCCACCGATCCACA; IFN-γ forward: TCTTCTTGGATATCTGGAGGAACTG; IFN-γ reverse: GAGATAATCTGGCTCTGCAGGATT.

### Enzyme-linked immunosorbent assay

Animals were anesthetized. Whole blood was collected from the eyes, incubated overnight at 4 °C, and centrifuged for 15 min at 1000 × g for supernatant analysis. Transcardiac perfusion with normal saline was performed immediately after blood collection. The brain was rapidly removed from the cranial cavity for further isolation of the hippocampus under a stereomicroscope. The hippocampus was homogenized in PBS (100 g tissue + 1 ml 1 × PBS) and stored overnight at − 20 °C. After two freeze‒thaw cycles were performed to break the cell membranes, the homogenate was centrifuged for 5 min at 5000 × g at 4 °C. The supernatant was obtained and assayed immediately. IFN-γ concentrations were determined using a mouse IFN-γ enzyme-linked immunosorbent assay (ELISA) kit (CUSABIO, China) according to the manufacturer’s instructions.

### CD8 + T-cell depletion

To deplete CD8 + T cells in vivo, mice were i.p. injected with 200 mg of anti-mouse CD8a (YTS 169.4, BioXcell, BE0117) twice 1 week prior to the surgery and once more the day after surgery. An equivalent amount of rat IgG2b (LTF-2, BioXcell, BE0090) was injected into the control group of animals.

### Hippocampal microinjections

Mice were anesthetized and fixed on a brain stereotaxic device with tribromoethanol, and then the skull was fully exposed. Bregma was used as the origin of the stereotaxic coordinates. The hippocampus was located according to the following stereotaxic coordinates: − 2.0 mm anterior–posterior, ± 2.5 mm medial–lateral, and − 2.0 mm dorsal–ventral. A tightly fitted screw was used to drill skull holes overlying the hippocampus on both hemispheres. A microsyringe was used to deliver bilateral intrahippocampal injections of IFN-γ neutralizing antibody (10 mg/ml, XMG1.2, BioXcell) or adeno-associated virus (AAV) (4.71e + 12 vg/ml, Beijing Syngentech Co., Ltd.). The control group was injected with the same amount of IgG1 isotype control or negative control AAV. A total volume of 1.0 µl was injected for no less than 5 min. The injection syringe was left in place for an additional 10 min to allow diffusion before it was slowly withdrawn. Pressure with gauze was applied to ensure adequate hemostasis, and then the incision was sutured layer by layer. The entire procedure strictly adhered to aseptic operation principles.

### AAV construction

Recombinant serotype 9 AAV that carried IFNGR1 shRNA (target sequence: 5′-GCCAGAGTTAAAGCTAAGGTT-3′; Syngentech, Beijing, China) was constructed. The target sequence for the negative control was 5′-AAACGTGACACGTTCGGAGAA-3′, which was used for mock transfection. Successful knockdown of IFNGR 1 was confirmed by western blotting 4 weeks after viral injection. To verify the location of injection, AAV-expressed EGFP was additionally constructed, and brain sections were taken 4 weeks after microinjection to observe the location of autofluorescence.

### Western blot analysis

Total protein was extracted by incubation in RIPA lysis buffer containing 1 mmol/L PMSF, and the protein concentrations were determined with a BCA Protein Assay Kit (Beyotime Biotechnology, China). The denatured proteins were separated using a 10% SDS‒PAGE gel and transferred to PVDF membranes using wet transfer at a 220 mA constant current. The membranes were blocked with 5% skim milk in TBST for 1 h at room temperature and incubated with primary antibodies (rabbit anti-IFNGR1 1:1000, Abcam; rabbit anti-beta-actin, 1:1000, Proteintech) at 4 °C overnight. On the following day, the membranes were incubated with secondary antibodies (goat anti-rabbit IgG–HRP, 1:5000, Proteintech) for 1 h at room temperature. The signals were detected by the chemiluminescence (ECL) method (Millipore Corporation, USA) and recorded using an Amersham Imager 680.

### Statistical analysis

Continuous variables were presented as the mean ± standard deviation. A two-tailed independent sample t test was applied to analyze the mean differences between two groups. One-way ANOVA was applied to compare means among multiple groups, and the LSD-t method was applied as a post hoc test. Repeated-measures ANOVA was applied for escape latency analysis and the swimming speed test. The Greenhouse–Geisser correction was used if the assumption of sphericity was not met. Quantification of cell number or band intensity was performed with ImageJ software (W. Rasband, NIH, USA). When counting the number of BrdU +, DCX + , DCX + /BrdU + or NeuN + /BrdU + cells, three standard hippocampal sections were randomly selected from each mouse, and the averages were used for statistical analysis. Statistical analyses were performed with SPSS 23.0 (SPSS Inc., Chicago, IL, USA). Graphs were made with GraphPad Prism Software version 8.0 (GraphPad Software, Inc., San Diego, CA, USA). All data were derived from three or more independent experiments. A value of *p* < 0.05 was considered to indicate statistical significance.

## Results

### Persistent cognitive decline induced by surgery

A flowchart of the experimental procedures is shown in Fig. 1A. No significant differences in oxygen partial pressure, lactic acid or hemoglobin were found among groups (Table 1). The MWM test and Y maze spontaneous alternation test were conducted to evaluate cognitive function after surgery. No significant differences in swimming speed or the number of total arm entries were observed among the groups, which excluded motor deficits (Fig. 1B, and C). The learning curve in the surgery group differed significantly from that in the control group at weeks 2, 3 or 4 after surgery (S2 vs. C, *p* < 0.001; S3 vs. C, *p* < 0.001; S4 vs. C, *p* < 0.001), showing persistent impaired spatial learning up to 4 weeks after surgery (Additional file 2: Figure S2 and Fig. 1D). The number of times crossing the previous platform location was significantly lower in the surgery group than in the control group (S2 vs. C, *p* = 0.007; S3 vs. C, *p* = 0.030; S4 vs. C, *p* = 0.015) (Additional file 2: Figure S2 and Fig. 1E). The mice in the S2 and S3 groups showed a significantly decreased dwell time in the target quadrant compared with the mice in the control group (S2 vs. C, *p* < 0.001; S3 vs. C, *p* < 0.001) (Additional file 2: Figure S2). Although not significant, a tendency toward decreased dwell times in the target quadrant was also observed in the S4 group (*p* = 0.065) (Fig. 1F). The Y-maze spontaneous alternation test was conducted on the day after the MWM test. Compared with the mice in the control group, mice in the surgery group also showed decreased rates of spontaneous alternation (S2 vs. C, *p* < 0.001; S3 vs. C, *p* = 0.007; S4 vs. C, *p* = 0.039) (Additional file 1: Figure S2 and Fig. 1G).

In addition, we compared the cognitive function of mice at different weeks after surgery, and mice that had undergone surgery showed a progressive improvement in the escape latency (S4 vs. S2, *p* = 0.006), dwell time in the target quadrant (S4 vs. S2, *p* = 0.039), and rates of spontaneous alternation (S4 vs. S2, *p* = 0.047) over time (Fig. 1H–K).

### Inhibition of AHN after surgery in adult mice

**Fig. 2:**
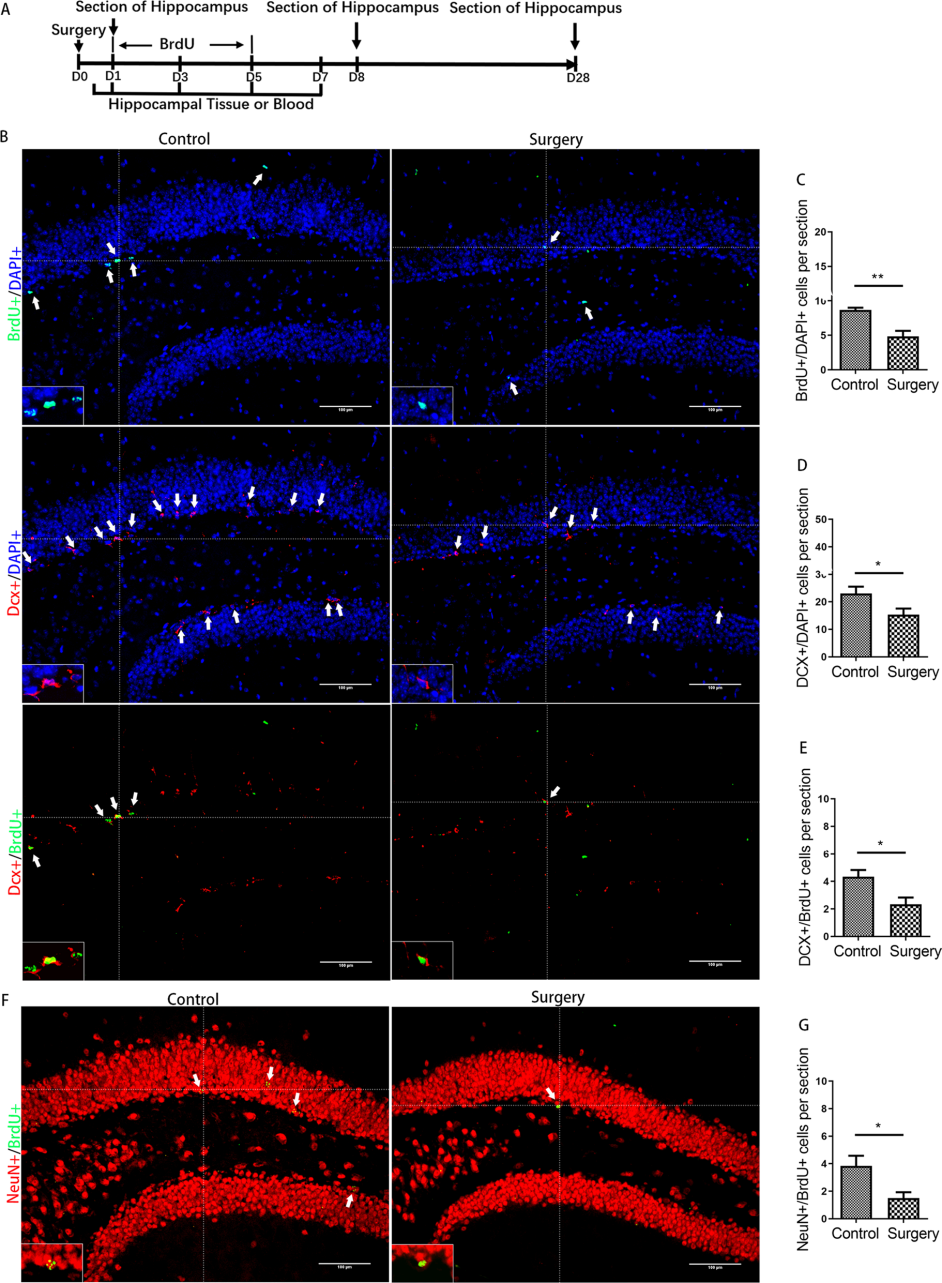
Surgery induced inhibition of neurogenesis in the hippocampus. **A** Experimental protocol diagram; **B** representative immunofluorescence images of BrdU +, Dcx + and Dcx + /BrdU + cells (200 ×); **C**–**E** quantitative analysis of BrdU +, Dcx + and Dcx + /BrdU + cells; **F** representative immunofluorescence images of NeuN + /BrdU + cells (200 ×); **G** quantitative analysis of NeuN + /BrdU + cells. Scale bar = 100 µm. The image at the bottom left showed a magnification of the crosshair position (400 ×). *N* = 6/group. D, day post operation. *p < 0.05, **p < 0.01

To evaluate whether the cognitive decline caused by surgery was related to AHN, BrdU was injected intraperitoneally for 5 days in succession after modeling, and hippocampal tissues were harvested for immunofluorescence staining to calculate the number of newborn cells (BrdU +), neuroblasts or immature neurons (DCX + , doublecortin +), newly generated neuroblasts or immature neurons (DCX + /BrdU +) and newly generated mature neurons (NeuN + /BrdU +) in the dentate gyrus (Fig. 2A). On day 8 after surgery, compared with the control group, the surgery group showed significantly decreased numbers of BrdU + cells, DCX + cells and DCX + /BrdU + cells (*p* = 0.004, *p* = 0.045, *p* = 0.017) (Fig. 2B–E). On day 28 after surgery, significantly decreased number of NeuN + /BrdU + cells was observed in the surgery group compared with the control group (*p* = 0.022) (Fig. 2F, and G).

### CD8 + T-cell infiltrated into the hippocampus after surgery

**Fig. 3:**
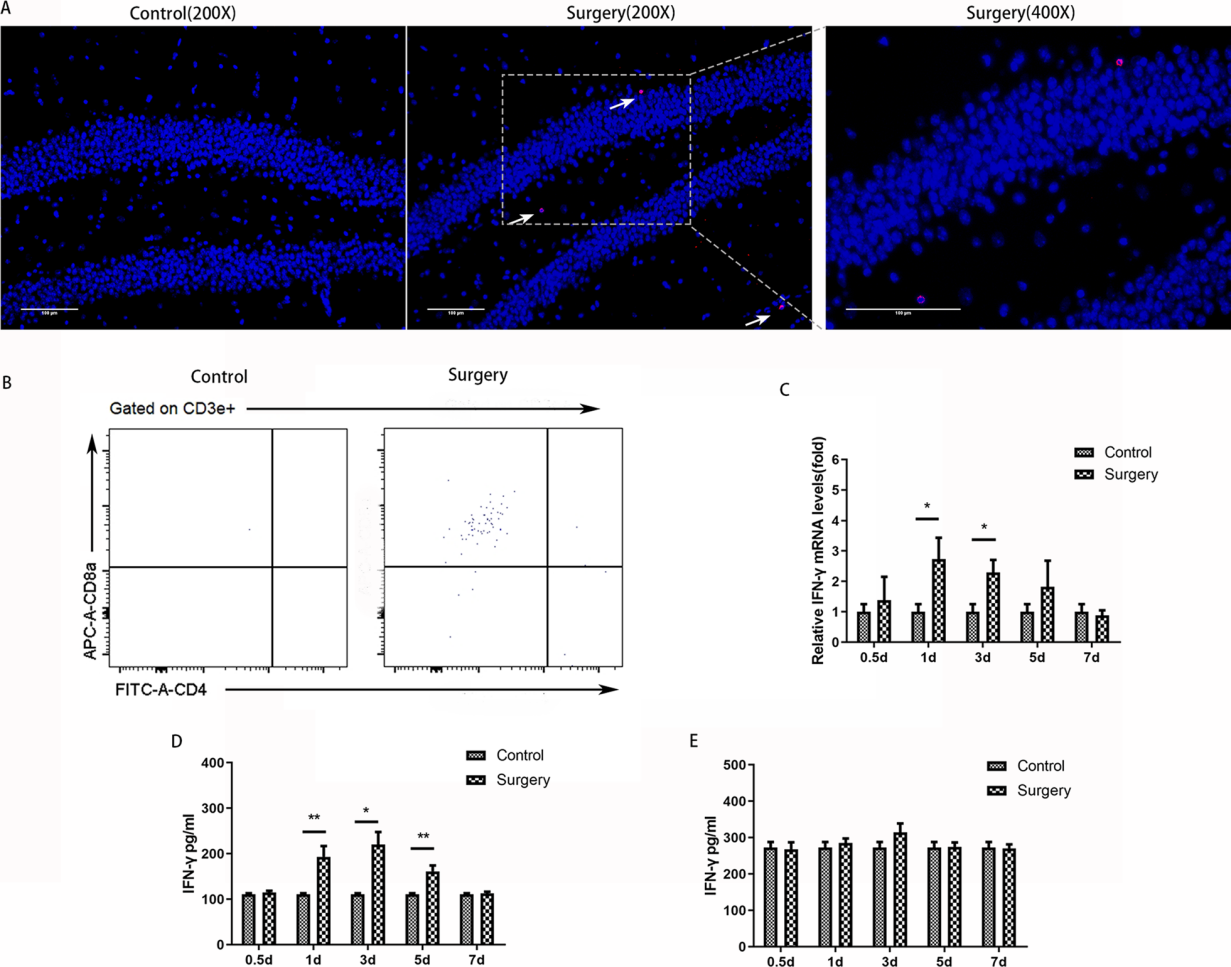
CD8 + T cells infiltrated the hippocampus and produced IFN-γ after surgery. **A** Representative immunofluorescence image of CD3 + T cells in the hippocampus of mice after surgery; **B** representative flow cytometry plots showing that the T cells infiltrating the hippocampus were mainly CD8 + T cells; **C** relative IFN-γ mRNA levels in the hippocampus of mouse, *N* = 5/group; **D** IFN-γ concentrations in the hippocampus, *N* = 5/group; **E** IFN-γ concentrations in plasma, *N* = 5/group. *p < 0.05, **p < 0.01

To observe whether T cells infiltrated the hippocampus after surgery, hippocampal tissues were harvested on the day following surgery for immunofluorescence staining of CD3, a marker of T cells. CD3-positive T-cell infiltration in the hippocampus was observed in the surgery group but not in the control group (Fig. 3A and Additional file 3: Figure S3). Furthermore, flow cytometry analysis showed that T cells infiltrating the hippocampus were mainly CD8 + T cells (Fig. 3B).

As IFN-γ is predominantly produced by T cells, we further obtained hippocampal tissue at 0.5, 1, 3, 5 or 7 days after surgery for real-time PCR and ELISA (Fig. 3C, D). The results showed that IFN-γ mRNA expression in the surgery group was significantly greater than that in the control group at 1 and 3 days after surgery (*p* = 0.048, *p* = 0.015). Increased IFN-γ levels in the hippocampus developed 1 day after surgery and persisted through postoperative day 5 (day 1, *p* = 0.009; day 3, *p* = 0.017; day 5, *p* = 0.006). For IFN-γ in the blood, there were no significant differences between the control group and surgery group at any time point (Fig. 3E).

### CD8 + T-cells infiltrating the hippocampus were related to the inhibition of AHN and late postoperative cognitive decline caused by surgery

**Fig. 4:**
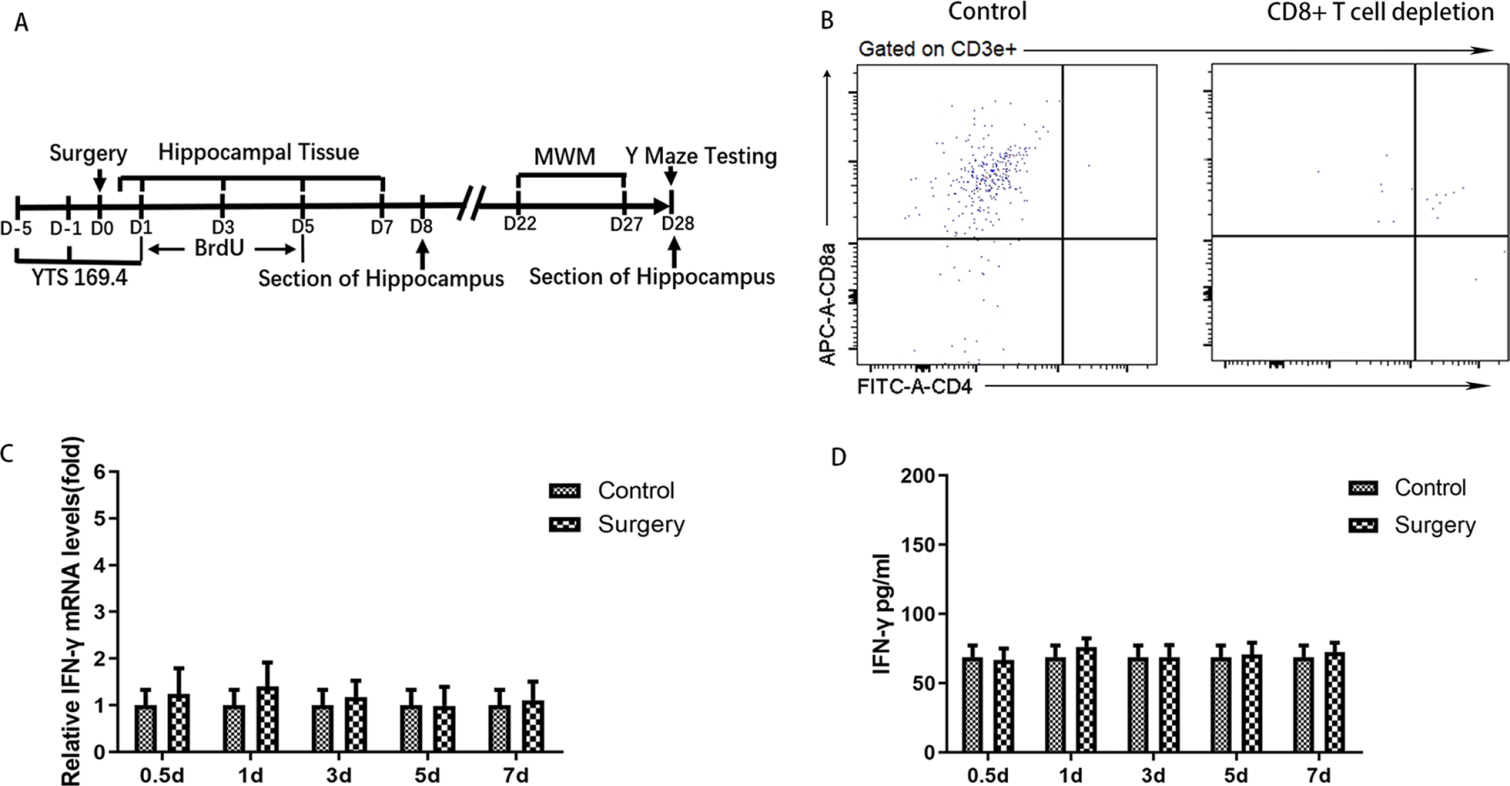
Administration of anti-CD8 antibody depleted CD8 + T cells. **A** Experimental protocol diagram; **B** representative flow cytometry plots showing CD8 + T-cell depletion; **C** relative IFN-γ mRNA levels in the hippocampus of mouse after surgery, *N* = 5/group; **D** IFN-γ concentrations in the hippocampus of mouse after surgery, *N* = 5/group. D, day post operation; MWM, morris water maze

**Fig. 5:**
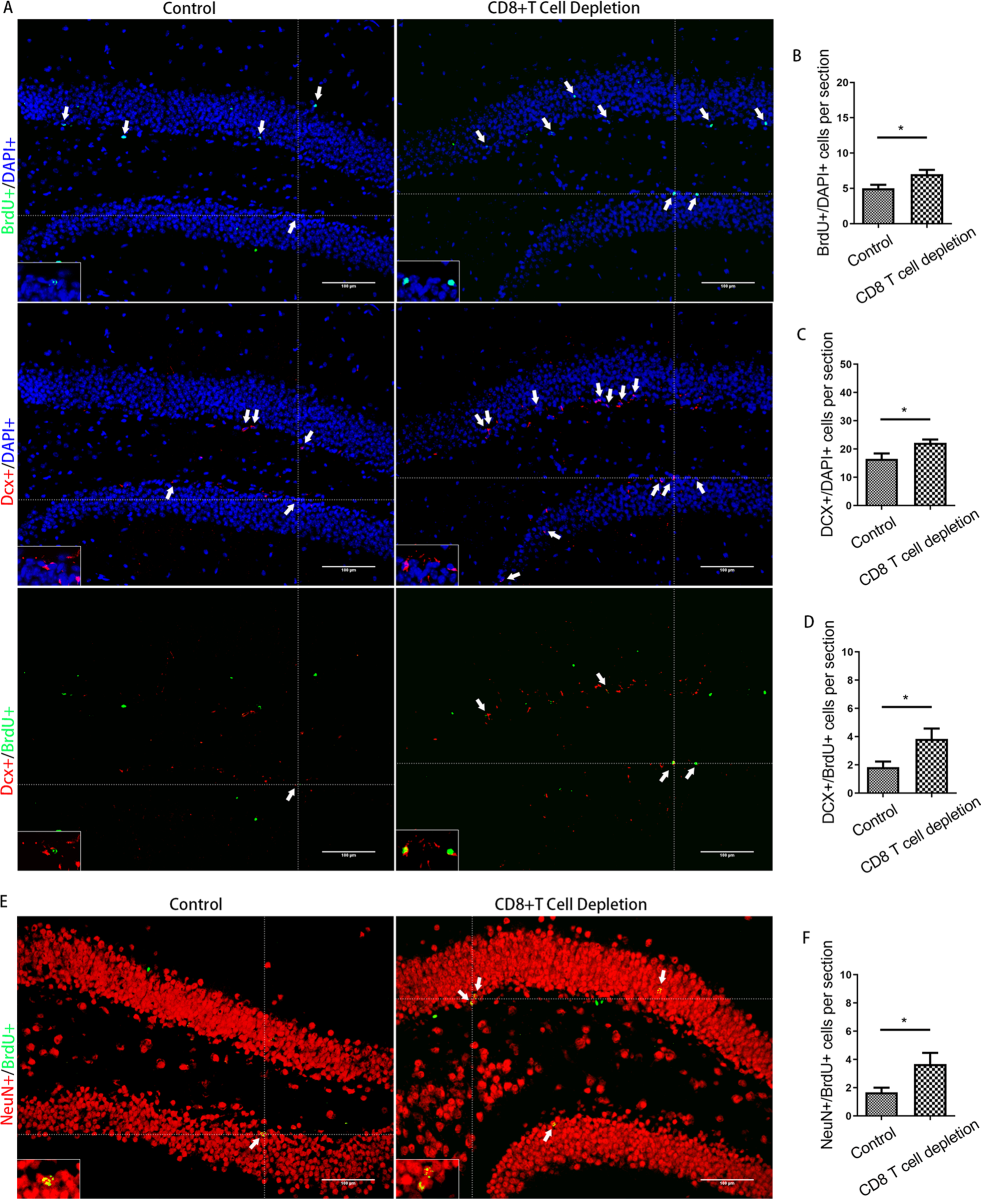
Inhibition of AHN induced by surgery could be partially prevented by administering anti-CD8 monoclonal antibodies. **A** Representative immunofluorescence images of BrdU +, Dcx + and Dcx + /BrdU + cells (200 ×); **B**–**D** quantitative analysis of BrdU + , Dcx + and Dcx + /BrdU + cells after surgery; **E** representative immunofluorescence images of NeuN + /BrdU + cells (200 ×); **F** quantitative analysis of NeuN + /BrdU + cells after surgery. Scale bar = 100 µm. The image at the bottom left showed a magnification of the crosshair position (400 ×). *N* = 6/group. *p < 0.05, **p < 0.01

**Fig. 6:**
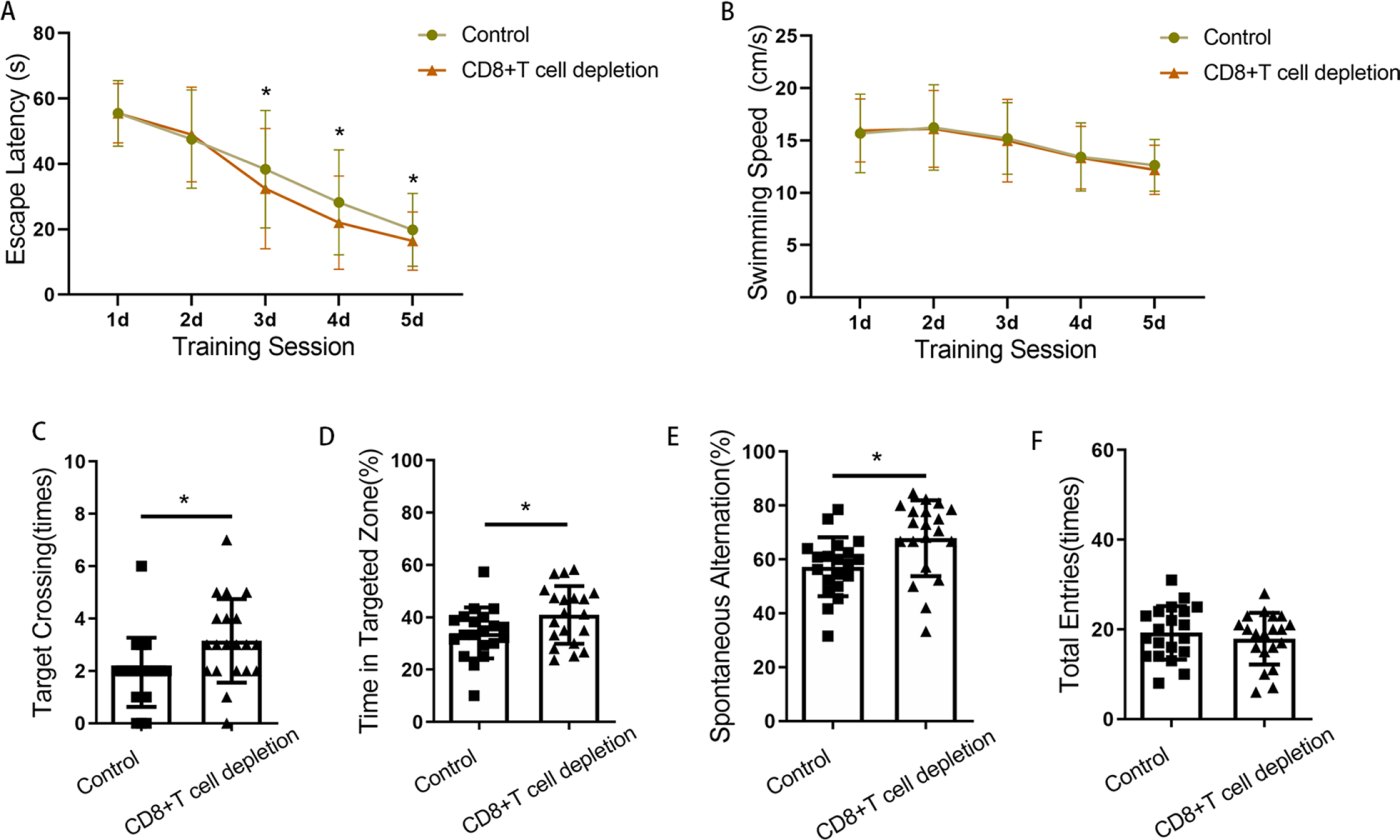
Cognitive impairment induced by surgery could be partially prevented by administering anti-CD8 monoclonal antibodies. **A** Escape latencies during training days in the MWM; **B** swimming speeds of mice during training days in the MWM; **C** times of target crossings on the testing day in the MWM; **D** percentages of time spent in the target zone on the testing day in the MWM; **E** spontaneous alternation rate in the Y maze; **F** the number of total arm entries in the Y maze. *N* = 20/group. *p < 0.05, **p < 0.01

Next, we asked whether CD8 + T cells were related to the inhibition of AHN and cognitive decline after surgery. A diagram of the experimental protocol is shown in Fig. 4A. When CD8 + T cells were depleted using a CD8a monoclonal antibody, a significant reduction in CD8 + T cells in the hippocampus was observed relative to the number in animals that received an isotype control (Fig. 4B). For mice with CD8 + T-cell depletion, there were no significant changes in either IFN transcript or protein levels after surgery (Fig. 4C, and D). For mice undergoing surgery, significantly improved nerve regeneration was observed following anti-CD8 monoclonal antibody vs. control IgG injection (for BrdU + cells, *p* = 0.035; for DCX + cells, *p* = 0.038; for DCX + /BrdU + cells, *p* = 0.048; for NeuN + /BrdU + cells, *p* = 0.044) (Fig. 5). Cognitive function was also improved, which specifically manifested as steeper learning curves (*p* = 0.019), more crossings of the platform position (*p* = 0.014), longer dwell times in the target quadrant (*p* = 0.043) and a higher rate of spontaneous alternation (*p* = 0.012) (Fig. 6). No significant differences in swimming speed and the number of total arm entries were observed among the groups.

### Blockade of IFN-γ/IFNGR could attenuate the inhibition of AHN and late postoperative cognitive decline caused by surgery

**Fig. 7:**
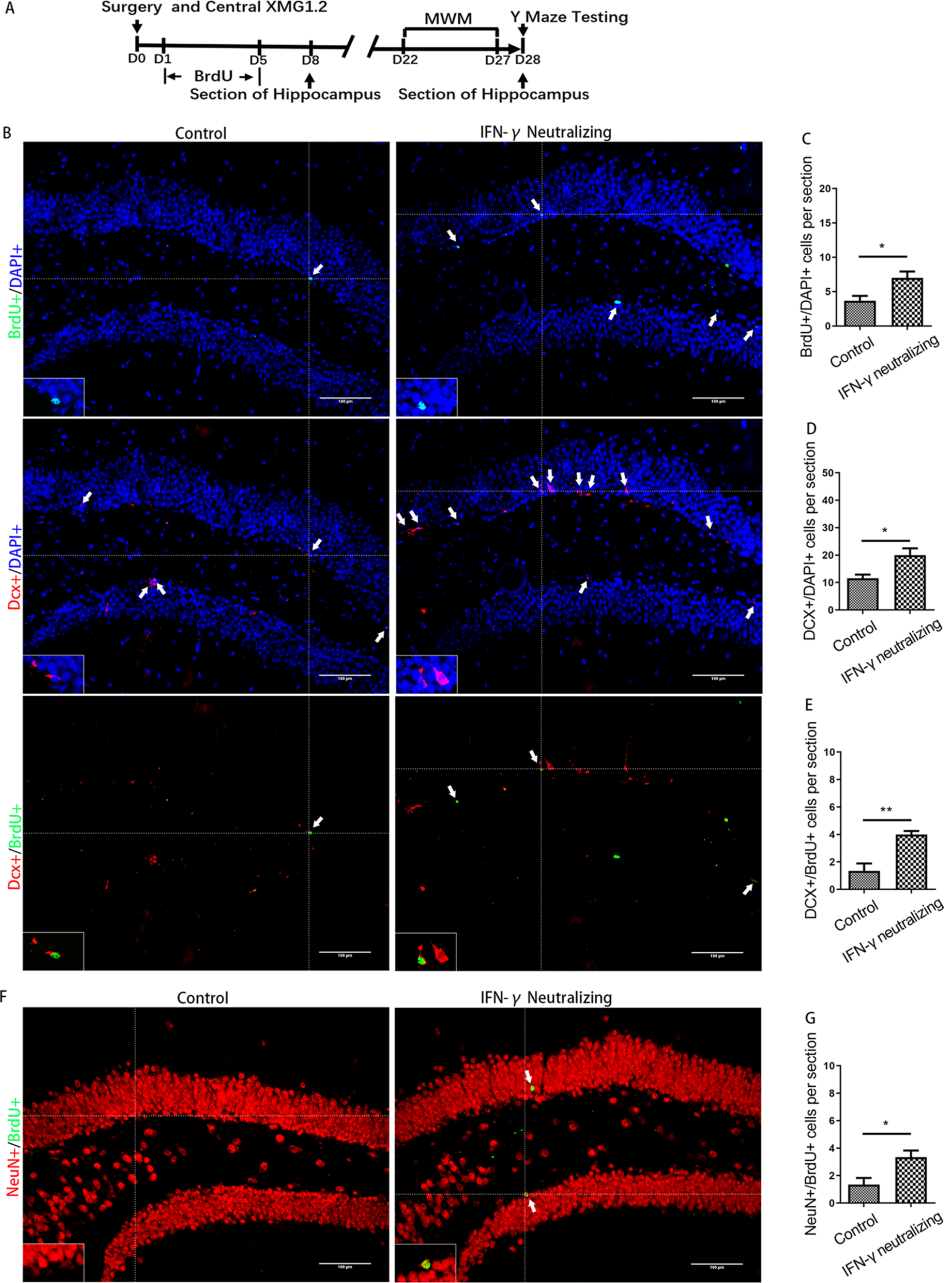
Inhibition of AHN induced by surgery could be partially prevented by IFN-γ neutralization. **A** Experimental protocol diagram; **B** representative immunofluorescence images of BrdU +, Dcx + and Dcx + /BrdU + cells (200 ×); **C**–**E** quantitative analysis of BrdU +, Dcx + and Dcx + /BrdU + cells; **F** representative immunofluorescence images of NeuN + /BrdU + cells (200 ×); **G** quantitative analysis of NeuN + /BrdU + cells. Scale bar = 100 µm. The image at the bottom left showed a magnification of the crosshair position (400 ×). *N* = 6/group. *p < 0.05, **p < 0.01

**Fig. 8:**
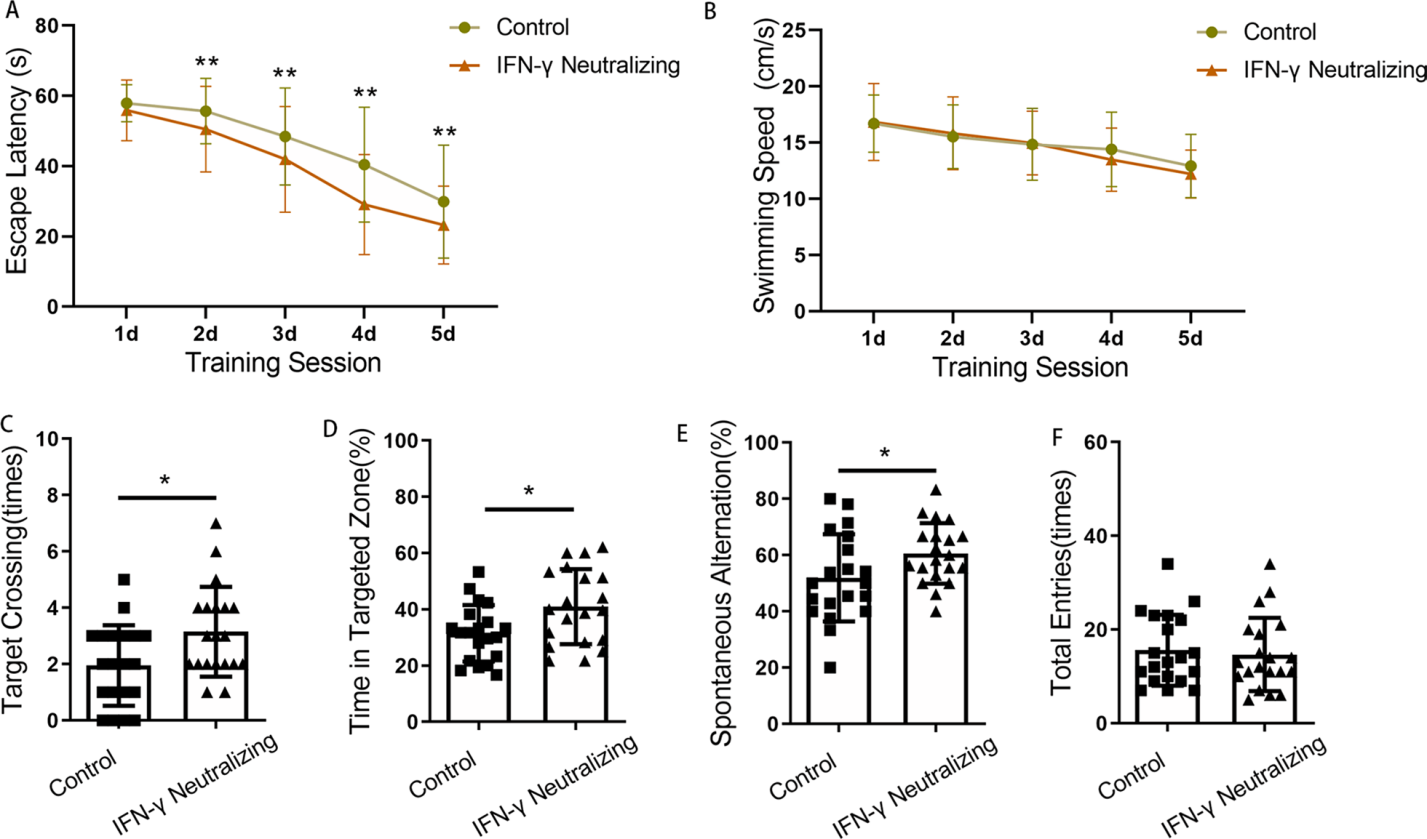
Cognitive impairment induced by surgery could be partially prevented by IFN-γ neutralization. **A** Escape latencies during training days in the MWM; **B** swimming speeds of mice during training days in the MWM; **C** times of target crossings on the testing day in the MWM; **D** percentages of time spent in the target zone on the testing day in the MWM; **E** spontaneous alternation rate in the Y maze; **F** number of total arm entries in the Y maze. *N* = 20/group. *p < 0.05, **p < 0.01

**Fig. 9:**
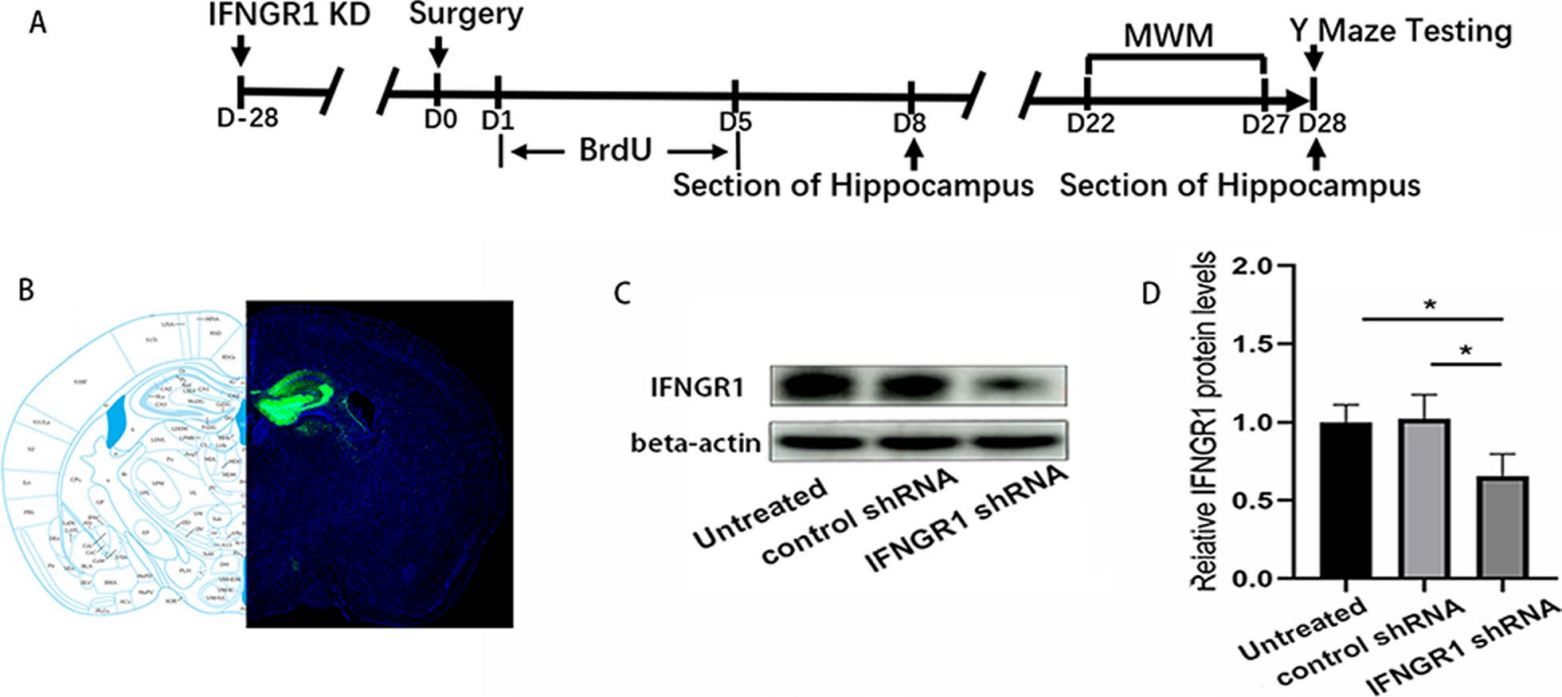
Knocking down IFNGR1. **A** Experimental protocol diagram after knockdown of IFNGR1; **B** representative EGFP fluorescence image of whole mouse brain section after AAV injection; **C** representative western blot image of IFNGR1 expression in the hippocampus; **D** relative expression of IFNGR1 protein, *N* = 3/group. D, day post operation; MWM, Morris water maze; KD, knockdown. *p < 0.05, **p < 0.01

To examine whether CD8 + T cells acted by secreting IFN-γ, IFN-γ/IFNGR was blocked by administering IFN-γ neutralizing antibodies or knocking down IFNGR1 (Figs. 7A, and 9A). Hippocampal microinjection of IFN-γ neutralizing antibodies attenuated the reductions in the numbers of BrdU + cells, DCX + cells, DCX + /BrdU + cells and NeuN + /BrdU + cells to some extent (*p* = 0.019, *p* = 0.018, *p* = 0.003 and *p* = 0.017) (Fig. 7B–G). In the behavioral tests at week 4 after surgery, mice with IFN-γ neutralizing antibodies showed steeper learning curves (*p* < 0.001), more crossings of the platform position (*p* = 0.017), longer dwell times in the target quadrant (*p* = 0.008) and a higher rate of spontaneous alternation (*p* = 0.047) (Fig. 8). No significant differences in swimming speed or the number of total arm entries were observed among the groups.

**Fig. 10:**
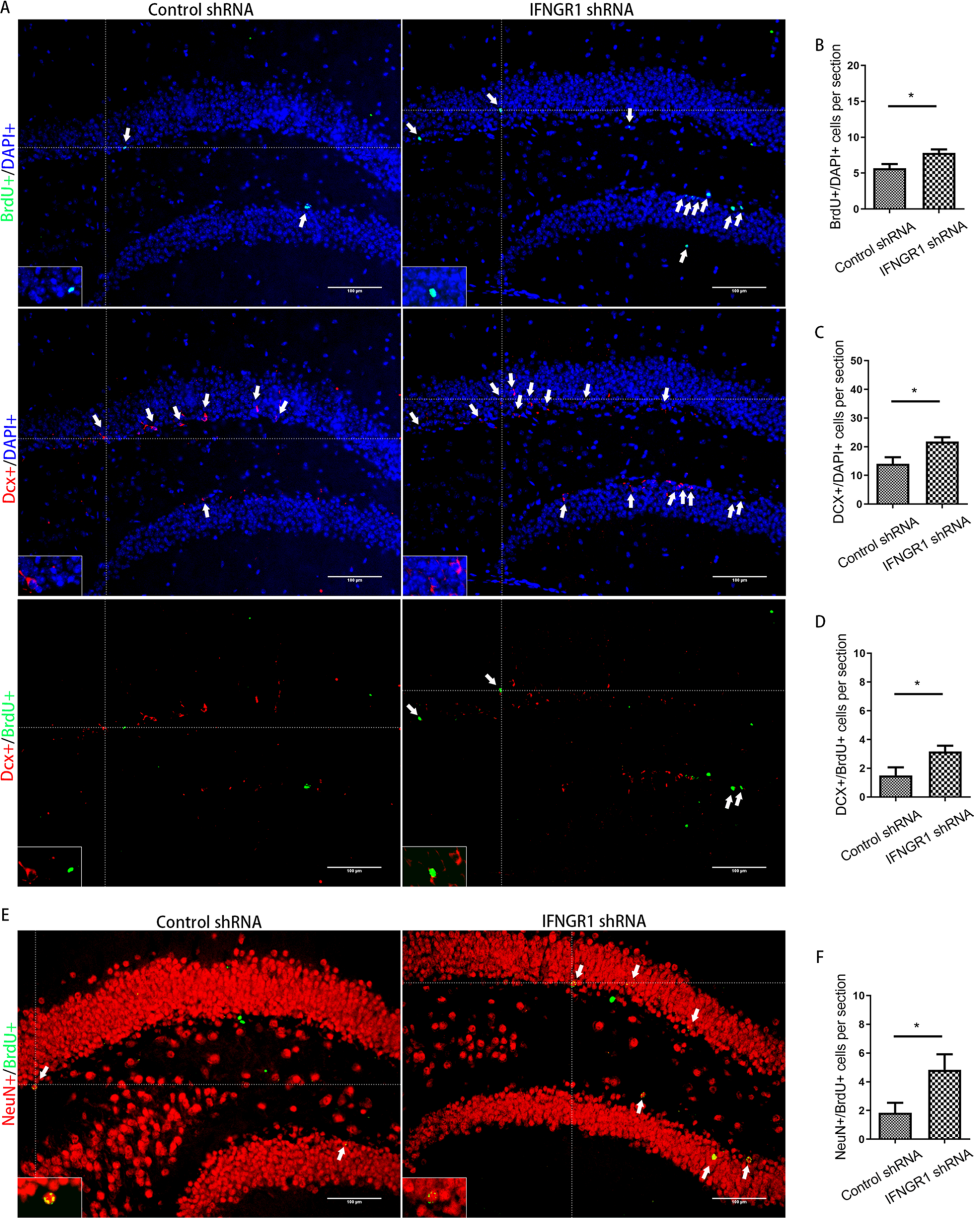
Inhibition of AHN induced by surgery could be partially prevented by silencing IFNGR1. **A** Representative immunofluorescence images of BrdU +, Dcx + and Dcx + /BrdU + cells (200 ×); **B**–**D** quantitative analysis of BrdU +, Dcx + and Dcx + /BrdU + cells after surgery; **E** representative immunofluorescence images of NeuN + /BrdU + cells (200 ×); **F** quantitative analysis of NeuN + /BrdU + cells after surgery. Scale bar = 100 µm. The image at the bottom left showed a magnification of the crosshair position (400 ×). *N* = 6/group. *p < 0.05, **p < 0.01

**Fig. 11:**
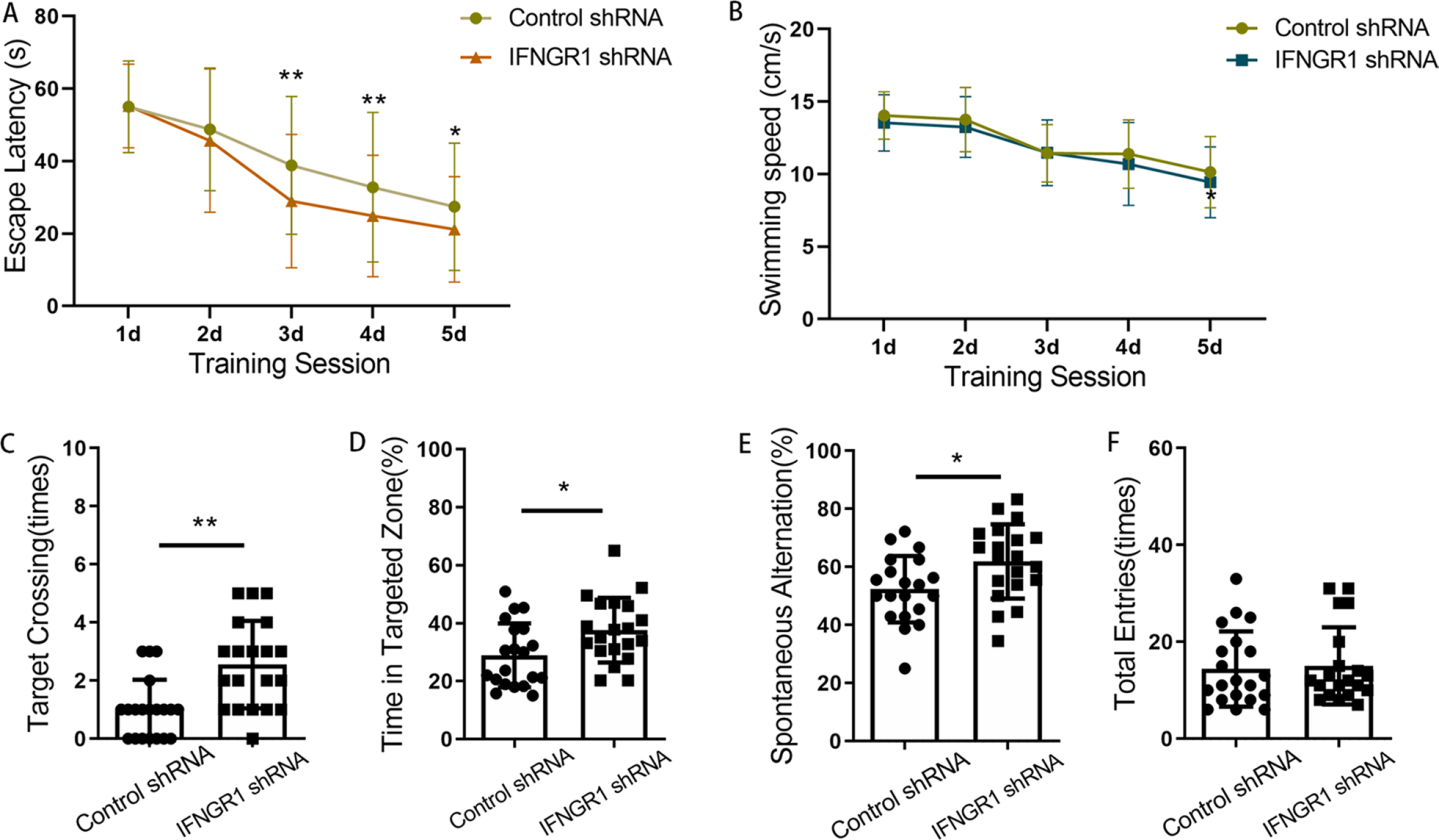
Cognitive impairment induced by surgery could be partially prevented by silencing IFNGR1. **A** Escape latencies during training days in the MWM; **B** swimming speeds of mice during training days in the MWM; **C** times of target crossings on the testing day in the MWM; **D** percentages of time spent in the target zone on the testing day in the MWM; **E** spontaneous alternation rate in the Y maze; **F** the number of total arm entries in the Y maze. *N* = 20/group. *p < 0.05, **p < 0.01

AAVs containing an IFNGR1 shRNA plasmid were subsequently constructed (Fig. 9A). Representative EGFP fluorescence image showed the location of virus infection after microinjection [28] (Fig. 9B). The downregulation of IFNGR1 protein was validated through western blotting 4 weeks after microinjection (Fig. 9C, D). IFNGR1 downregulation partially attenuated the AHN inhibition induced by surgery compared with that in the control group. Representative images of BrdU + cells (*p* = 0.048), DCX + cells (*p* = 0.001), DCX + /BrdU + cells (*p* = 0.033) and NeuN + /BrdU + cells (*p* = 0.042) were shown in Fig. 10. Compared with control mice, IFNGR1-knockdown mice exhibited steeper learning curves (*p* = 0.002), more crossings of the platform position (*p* = 0.001), longer dwell times in the target quadrant (*p* = 0.018) and a higher rate of spontaneous alternation (*p* = 0.018) after surgery (Fig. 11). There were no significant differences in swimming speed or the number of total arm entries.

## Discussion

In this study, we demonstrated that cognitive decline persisted in mice who underwent surgery during the first postoperative month, even though there was a trend toward continuous improvement over time. Surgery can significantly inhibit neurogenesis in the dentate gyrus of the hippocampus. After surgery, T lymphocytes, the majority of which were CD8 + T cells, infiltrated the hippocampus and secreted IFN-γ. Depletion of CD8 + T cells could inhibit the increase of IFN-γ synthesis, improve hippocampal neurogenesis, and improve postoperative cognitive function. Hippocampal microinjection of IFN-γ neutralizing antibody or adeno-associated virus knocking down IFNGR1 could also partially attenuate the inhibition of AHN and improve cognitive function later following surgery. These findings suggested that surgical trauma led to persistent cognitive decline for up to 1 month after surgery, which was associated with inhibition of AHN induced by CD8 + T-cell infiltration following surgery.

Many clinical studies have shown that postoperative cognitive decline is closely related to the type of surgery and is more common in patients who undergo major surgery, such as vascular, cardiac, abdominal, or hip fracture surgery, than in those who undergo minor surgery [29]. Exploratory laparotomy, partial hepatectomy and tibial fracture models have been widely used in research on postoperative cognitive decline [30–34]. In this study, to increase the incidence of postoperative cognitive decline, we adopted a surgical model of exploratory laparotomy combined with partial hepatectomy. There were no significant differences in oxygen partial pressure, lactic acid or hemoglobin among groups, which indicated that cognitive changes were likely not due to the effects of cerebral hypoxia or hypoperfusion caused by anesthesia/surgery.

The duration of postoperative cognitive decline depends on age, species, surgical procedure, perioperative medication use and other factors. Clinical studies have shown that cognitive decline can still occur weeks, months or even years after surgery [3]. Cognitive decline later following surgery has also been reported in animal models. Fidalgo et al. found that the remote memory of mice was significantly impaired 26 days after peripheral orthopedic surgery [35]. It has also been reported that memory impairment in aged rats that underwent laparotomy combined with morphine analgesia could last for at least 2 months [31]. Our results showed that even though there was a trend toward continuous improvement over time, cognitive decline persisted in mice during the first month after surgery, which was consistent with clinical observations. However, conflicting results showed that spatial learning and memory were temporarily impaired in the first 2 weeks following abdominal surgery but restored at 3 weeks in rats [6]. The difference in conclusions may be partly attributable to the different types of operation, severity of trauma, ages of the experimental animals and insufficient sample sizes.

The dentate gyrus in the hippocampus is responsible for spatial navigation and cognitive functions. It receives signals from the entorhinal cortex and transmits them to other areas of the hippocampus. Neural stem cells in the SGZ of the dentate gyrus continue to proliferate and differentiate, constantly adding new neurons to the granular layer and integrating into the neural network. Hippocampal neurogenesis persists throughout life in the brains of humans and rodents and is the most robust form of plasticity in the adult brain [8, 9]. Substantial literature has confirmed that AHN is closely related to neurocognitive function and a decline of AHN contributes to several human neurological and psychiatric diseases, such as Alzheimer’s disease, depression, and age-associated cognitive decline [9, 11, 13]. Inhalation anesthesia in early life can decrease neurogenesis in the hippocampus and may be implicated in long-term learning and memory dysfunction during adulthood [36]. We previously reported that inhibition of AHN played an important role in cognitive decline 4 weeks after sevoflurane exposure in aged mice through the BDNF/TrkB and NT-3/TrkC pathways [37].

Before functionally integrating into the neural circuitry, adult-born dentate granule neurons in the hippocampus pass through several consecutive developmental stages, including transient amplification of neural stem cells, giving rise to neuroblasts and subsequently differentiating into mature neurons [38]. The developmental trajectory is accompanied by subsequent expression of stage-specific molecular markers. When intermediate neural progenitors differentiate into neuroblasts, they gain expression of DCX, which is expressed continuously before maturation. BrdU is used to label proliferating cells, as it can be incorporated instead of thymidine into newly synthesized DNA. Our results showed significant decreases in DCX + cells, BrdU + cells, and BrdU + /DCX + cells on day 8 after surgery. In accordance with our results, decreased DCX +, BrdU +, or BrdU + /DCX + staining has been reported following several surgery models, such as ischemia–reperfusion of the upper mesenteric artery, ischemia–reperfusion of the left coronary artery, and carotid exposure [6, 39–41]. Notably, the decrease in neurogenesis appears to be independent of the type of surgery, as it was reported that there was no significant difference in the postoperative number of DCX-positive cells between animals that underwent cardiac surgery and those that underwent abdominal surgery [39].

Moreover, a long period is needed for immature neurons (Dcx + cells) to develop into mature granular neurons, during which immature neurons undergo a series of metabolic, morphological, and functional changes under the influence of internal and external environments [38]. From the second week of development, newborn neurons successively receive inputs from hilar mossy cells, inhibitory interneurons in the SGZ and the hilus, cells in the molecular layer, the entorhinal cortex, back-projection from the CA3, mature granule cells and the subiculum [42–44]. Axons are found in the hilus as early as 7 days after infection with GFP-expressing retroviruses and reach the CA3 at 10–11 days, where they form functional glutamatergic connections 17 day post-mitosis [45]. By the end of the fourth week, newborn neurons are already integrated into the circuitry of the hippocampus, and their morphological growth is mostly complete. Newborn neurons are more excitable and have enhanced synaptic plasticity, which allows them to respond to a broad range of input stimuli and quickly reinforce active connections [45–47]. NeuN is a marker of mature neurons. Few reports have described postoperative changes in the number of BrdU + /NeuN + cells. Our results showed that the BrdU + /NeuN + cell population in the dentate gyrus of the hippocampus was significantly reduced at 28 days following surgery. These results indicate that surgical trauma could inhibit AHN.

AHN is regulated by many factors, among which neuroinflammation plays an important role. Our results suggested that the adaptive immune response mediated by T cells may play an important role in this process. It is ever reported CD8 + T-cell infiltration into the brain starts around 18 months of age in WT mice. However, in some pathological states, T-cell infiltration can be detected in the brains of younger mice [48]. T cells may infiltrate the brain through the leakier blood–brain barrier or the recently discovered meningeal lymphatic vessels after surgery [49, 50]. It has been previously reported that CNS (central nervous system) myeloid cells are activated and act as antigen-presenting cells to mediate T cells entry into the parenchyma during neuroinflammation. The upregulation of MHC-I class molecules in CNS myeloid cells is necessary for the infiltration of T cells into the hippocampus [51]. Our previous RNA sequencing results also suggested increased expression of the H2 complex in the hippocampus after surgery (Additional file 1: Figure S1). Our previous study also reported activation of microglia specified by the transition from a highly branched lace morphology to one with shortened processes, enlarged cell bodies and increased expression of Iba1 in the hippocampus after surgery [52]. However, T-cell infiltration into the hippocampus is a complex process. In addition to antigen presentation, chemokines, chemokine receptors and adhesion molecules may also play important roles. In addition, there may be crosstalk between T cells and microglia after surgery. It has been reported that T cells in the CNS can secrete IFN-γ, which is a potent activator of microglia, in antigen-dependent or nonantigen-dependent form [17, 53, 54]. The results of our current study also showed increased IFN-γ synthesis in the hippocampus after surgery. Therefore, the mechanism of T-cell infiltration into the hippocampus after surgery needs to be investigated by more rigorous scientific experiments.

Different chemokines, different antigens and different levels of blood–brain barrier disruption may control the movement of distinct subsets of immune cells into specific tissues. In this study, we reported that T cells infiltrating the hippocampus after surgery were mainly CD8 + T cells. Postoperative infiltration of CD8 + T cells into the hippocampus may be involved in the pathological process of AHN inhibition and cognitive decline, as our results showed that depletion of CD8 + T cells could improve postoperative AHN and cognitive function. Antigen recognition is critical for the general damage induced by CD8 + T cells. With aging, self-antigens, such as amyloid precursor protein, amyloid-β, tau, α-synuclein and transactive response DNA binding protein, accumulate gradually in the brain and can be specifically recognized by T cells and trigger an immune response [50]. Increased expression of MHC-I in central neurons with age has also been reported previously [15]. These findings may help explain why older individuals are more susceptible to the development of cognitive function decline after surgery.

One of the major effector functions of CD8 + T cells is the secretion of IFN-γ. Our results show that increased IFN-γ levels in the hippocampus after surgery may originate from central CD8 + T cells rather than peripheral blood. This is consistent with previous reports. There was no significant increase in IFN-γ in the blood of patients who had undergone major knee or hip replacement [22, 55]. For mice undergoing exploratory laparotomy, peripheral CD8 + T-cell function was inhibited, and IFN-γ secretion was reduced [56]. Increased IFN-γ has been shown to directly interact with the cell-surface IFNGR of neural stem cells or immature neurons, and then inhibit neural stem cell proliferation and reduce neurogenesis [23]. The absence of IFN-γ led to increases in the numbers of newborn neurons in the SGZ of the hippocampus and enhanced cognitive behaviors in tasks involving the hippocampus [57]. IFN-γ can also act on other cells, such as microglia, to regulate the innate immune response. Substantial IFNGR is present on the surface of microglia [17]. Adding a low dose of IFN-γ into hippocampal slice cultures could induce substantial proliferation and moderate activation of microglia and then lead to a specific decline in the frequency of gamma oscillations, which are fundamental to higher brain functions, such as perception, attention, and memory [58]. Activated microglia can release a variety of inflammatory mediators, contributing to reduced neurogenesis [59]. Mice that received intracerebroventricular injections of IFN-γ showed a significant reduction in hippocampal neurogenesis as well as cognitive decline and depressive-like behaviors [60]. Microglia isolated from the hippocampus of IFN-γ-injected mice suppressed the proliferation of neural stem/precursor cells and stimulated apoptosis of immature neurons [60]. Our results showed that the surgery-induced decreases in DCX + cell, BrdU + cell, BrdU + /DCX + cell and BrdU + /NeuN + cell were partially attenuated by administering IFN-γ neutralizing antibody or knocking down IFNGR1 in the hippocampus.

Given that numerous spines that are essential for information transmission are formed during the third-to-fourth weeks of neonatal neuron development [45–47], we examined the changes of cognitive function in mice during the fourth week following surgery after administering anti-CD8 monoclonal antibody, IFN-γ neutralizing antibody or knocking down IFNGR1. The results showed that cognitive decline later following surgery was also partially ameliorated. It is undeniable that the incidence of cognitive decline after surgery progressively decreases with time with the resolution of acute inflammation, with compensatory enhancement of synaptic plasticity or for other reasons [14, 31]. However, for patients with poor capacity for autoregulation and compensation partly due to advanced age, encephalopathy, severe trauma, or postoperative complications, the influence of AHN inhibition appeared to be particularly important.

This study has some limitations. First, we focused only on spatial learning and memory functions, the main aspects of cognitive dysfunction. However, cognitive dysfunction also includes many other aspects. Second, the specific mechanism by which CD8 + T cells infiltrated the hippocampus was not elucidated. Third, we were not able to identify which stages of AHN were suppressed.

In conclusion, the present results showed that surgical trauma can inhibit AHN, leading to cognitive decline later following surgery, and postoperative infiltration of CD8 + T cells into the hippocampus and subsequent secretion of IFN-γ played important roles in this pathophysiological process. Identifying viable therapeutic strategies to tackle abnormal infiltration of CD8 + T cells may provide effective interventions for cognitive decline later following surgery.

### Supplementary Information

**Additional file 1: Figure S1.** RNA sequencing of the hippocampus suggested the possibility of leukocyte infiltration after surgery. (A) GO enrichment analysis; (B) KEGG pathway analysis.

**Additional file 2: Figure S2.** Learning and memory performance at week 2 and week 3 after surgery. (A), (E) Escape latencies during training days; (B), (F) Times of target crossings on the testing day; (C), (G) Percentages of time spent in the targeted zone on the testing day; (D), (H) Spontaneous alternation rate in the Y maze. C, control; S2/S3, animals that underwent behavioral testing during weeks 2/3 after surgery. *N* = 16–20/group. * *p* < 0.05, ** *p* < 0.01.

**Additional file 3: Figure S3.** Representative immunofluorescence image of CD3 + T cells in the hippocampus of mice after surgery.

## Data Availability

The data sets used and/or analyzed during the current study are available from the corresponding author on reasonable request.

## Abbreviations

AHN: Adult hippocampal neurogenesis
IFN-γ: Interferon-γ
IFNGR1: Interferon-γ receptor 1
POD: Postoperative delirium
DNR: Delayed neurocognitive recovery
pNCD: Postoperative neurocognitive disorder
SGZ: Subgranular zone
MWM: Morris water maze
BrdU: Bromodeoxyuridine
ELISA: Enzyme-linked immunosorbent assay
AAV: Adeno-associated virus
DCX: Doublecortin

## References

[1] Evered L, Silbert B, Knopman DS, Scott DA, DeKosky ST, Rasmussen LS, Oh ES, Crosby G, Berger M, Eckenhoff RG (2018). Recommendations for the nomenclature of cognitive change associated with anaesthesia and surgery-2018. Anesthesiology.

[2] Daiello LA, Racine AM, Yun Gou R, Marcantonio ER, Xie Z, Kunze LJ, Vlassakov KV, Inouye SK, Jones RN, Alsop D (2019). Postoperative delirium and postoperative cognitive dysfunction: overlap and divergence. Anesthesiology.

[3] Monk TG, Weldon BC, Garvan CW, Dede DE, van der Aa MT, Heilman KM, Gravenstein JS (2008). Predictors of cognitive dysfunction after major noncardiac surgery. Anesthesiology.

[4] McDonagh DL, Mathew JP, White WD, Phillips-Bute B, Laskowitz DT, Podgoreanu MV, Newman MF (2010). Cognitive function after major noncardiac surgery, apolipoprotein E4 genotype, and biomarkers of brain injury. Anesthesiology.

[5] Deiner S, Liu X, Lin HM, Jacoby R, Kim J, Baxter MG, Sieber F, Boockvar K, Sano M (2021). Does postoperative cognitive decline result in new disability after surgery?. Ann Surg.

[6] Hovens IB, Schoemaker RG, van der Zee EA, Absalom AR, Heineman E, van Leeuwen BL (2014). Postoperative cognitive dysfunction: involvement of neuroinflammation and neuronal functioning. Brain Behav Immun.

[7] Barrientos RM, Hein AM, Frank MG, Watkins LR, Maier SF (2012). Intracisternal interleukin-1 receptor antagonist prevents postoperative cognitive decline and neuroinflammatory response in aged rats. J Neurosci.

[8] Boldrini M, Fulmore CA, Tartt AN, Simeon LR, Pavlova I, Poposka V, Rosoklija GB, Stankov A, Arango V, Dwork AJ (2018). Human hippocampal neurogenesis persists throughout aging. Cell Stem Cell.

[9] Tobin MK, Musaraca K, Disouky A, Shetti A, Bheri A, Honer WG, Kim N, Dawe RJ, Bennett DA, Arfanakis K, Lazarov O (2019). Human hippocampal neurogenesis persists in aged adults and Alzheimer’s disease patients. Cell Stem Cell.

[10] Moreno-Jiménez EP, Flor-García M, Terreros-Roncal J, Rábano A, Cafini F, Pallas-Bazarra N, Ávila J, Llorens-Martín M (2019). Adult hippocampal neurogenesis is abundant in neurologically healthy subjects and drops sharply in patients with Alzheimer’s disease. Nat Med.

[11] Du Preez A, Onorato D, Eiben I, Musaelyan K, Egeland M, Zunszain PA, Fernandes C, Thuret S, Pariante CM (2021). Chronic stress followed by social isolation promotes depressive-like behaviour, alters microglial and astrocyte biology and reduces hippocampal neurogenesis in male mice. Brain Behav Immun.

[12] Gontier G, Iyer M, Shea JM, Bieri G, Wheatley EG, Ramalho-Santos M, Villeda SA (2018). Tet2 rescues age-related regenerative decline and enhances cognitive function in the adult mouse brain. Cell Rep.

[13] Leiter O, Zhuo Z, Rust R, Wasielewska JM, Grönnert L, Kowal S, Overall RW, Adusumilli VS, Blackmore DG, Southon A (2022). Selenium mediates exercise-induced adult neurogenesis and reverses learning deficits induced by hippocampal injury and aging. Cell Metab.

[14] Needham MJ, Webb CE, Bryden DC (2017). Postoperative cognitive dysfunction and dementia: what we need to know and do. Br J Anaesth.

[15] Zhou L, Kong G, Palmisano I, Cencioni MT, Danzi M, De Virgiliis F, Chadwick JS, Crawford G, Yu Z, De Winter F (2022). Reversible CD8 T cell-neuron cross-talk causes aging-dependent neuronal regenerative decline. Science.

[16] Altendorfer B, Unger MS, Poupardin R, Hoog A, Asslaber D, Gratz IK, Mrowetz H, Benedetti A, de Sousa DMB, Greil R (2022). Transcriptomic profiling identifies CD8(+) T cells in the brain of aged and Alzheimer’s disease transgenic mice as tissue-resident memory T cells. J Immunol.

[17] Dulken BW, Buckley MT, Navarro Negredo P, Saligrama N, Cayrol R, Leeman DS, George BM, Boutet SC, Hebestreit K, Pluvinage JV (2019). Single-cell analysis reveals T cell infiltration in old neurogenic niches. Nature.

[18] Stojić-Vukanić Z, Hadžibegović S, Nicole O, Nacka-Aleksić M, Leštarević S, Leposavić G (2020). CD8+ T cell-mediated mechanisms contribute to the progression of neurocognitive impairment in both multiple sclerosis and Alzheimer’s disease?. Front Immunol.

[19] Sulzer D, Alcalay RN, Garretti F, Cote L, Kanter E, Agin-Liebes J, Liong C, McMurtrey C, Hildebrand WH, Mao X (2017). T cells from patients with Parkinson’s disease recognize α-synuclein peptides. Nature.

[20] Li Z, Mo N, Li L, Cao Y, Wang W, Liang Y, Deng H, Xing R, Yang L, Ni C (2016). Surgery-induced hippocampal angiotensin II elevation causes blood-brain barrier disruption via MMP/TIMP in aged rats. Front Cell Neurosci.

[21] He HJ, Wang Y, Le Y, Duan KM, Yan XB, Liao Q, Liao Y, Tong JB, Terrando N, Ouyang W (2012). Surgery upregulates high mobility group box-1 and disrupts the blood-brain barrier causing cognitive dysfunction in aged rats. CNS Neurosci Ther.

[22] Hirsch J, Vacas S, Terrando N, Yuan M, Sands LP, Kramer J, Bozic K, Maze MM, Leung JM (2016). Perioperative cerebrospinal fluid and plasma inflammatory markers after orthopedic surgery. J Neuroinflammation.

[23] Pereira L, Medina R, Baena M, Planas AM, Pozas E (2015). IFN gamma regulates proliferation and neuronal differentiation by STAT1 in adult SVZ niche. Front Cell Neurosci.

[24] Feng X, Valdearcos M, Uchida Y, Lutrin D, Maze M, Koliwad SK (2017). Microglia mediate postoperative hippocampal inflammation and cognitive decline in mice. JCI Insight.

[25] Flurkey K, Currer JM, DE H. Chapter 20—Mouse Models in Aging Research. The Mouse in Biomedical Research, 2nd Edition. Elsevier; 2007. p. 637–672.

[26] Dutta S, Sengupta P (2016). Men and mice: relating their ages. Life Sci.

[27] Wang S, Lai X, Deng Y, Song Y (2020). Correlation between mouse age and human age in anti-tumor research: significance and method establishment. Life Sci.

[28] Franklin KBJ, Paxinos G. The Mouse Brain in Stereotaxic Coordinates, The coronal plates and diagrams Compact, 3rd Edition. Elsevier; 2008. p. 44.

[29] Li Z, Zhu Y, Kang Y, Qin S, Chai J (2022). Neuroinflammation as the underlying mechanism of postoperative cognitive dysfunction and therapeutic strategies. Front Cell Neurosci.

[30] Liu Q, Liu L, Liu H, Jiang J, Guo S, Wang C, Jia Y, Tian Y (2019). Compound K attenuated hepatectomy-induced post-operative cognitive dysfunction in aged mice via LXRα activation. Biomed Pharmacother.

[31] Muscat SM, Deems NP, D'Angelo H, Kitt MM, Grace PM, Andersen ND, Silverman SN, Rice KC, Watkins LR, Maier SF, Barrientos RM (2021). Postoperative cognitive dysfunction is made persistent with morphine treatment in aged rats. Neurobiol Aging.

[32] Sun L, Yong Y, Wei P, Wang Y, Li H, Zhou Y, Ruan W, Li X, Song J (2022). Electroacupuncture ameliorates postoperative cognitive dysfunction and associated neuroinflammation via NLRP3 signal inhibition in aged mice. CNS Neurosci Ther.

[33] Wei P, Zheng Q, Liu H, Wan T, Zhou J, Li D, Zhou H, Li J, Ji F, Tang W, Li J (2018). Nicotine-induced neuroprotection against cognitive dysfunction after partial hepatectomy involves activation of BDNF/TrkB signaling pathway and inhibition of NF-κB signaling pathway in aged rats. Nicotine Tob Res.

[34] Cibelli M, Fidalgo AR, Terrando N, Ma D, Monaco C, Feldmann M, Takata M, Lever IJ, Nanchahal J, Fanselow MS, Maze M (2010). Role of interleukin-1beta in postoperative cognitive dysfunction. Ann Neurol.

[35] Fidalgo AR, Cibelli M, White JP, Nagy I, Noormohamed F, Benzonana L, Maze M, Ma D (2011). Peripheral orthopaedic surgery down-regulates hippocampal brain-derived neurotrophic factor and impairs remote memory in mouse. Neuroscience.

[36] Jia J, Zhu J, Yang Q, Wang Y, Zhang Z, Chen C (2020). The role of histone acetylation in the sevoflurane-induced inhibition of neurogenesis in the hippocampi of young mice. Neuroscience.

[37] Xu L, Guo Y, Wang G, Sun G, Sun W, Li J, Li X, Wu J, Zhang M (2022). Inhibition of adult hippocampal neurogenesis plays a role in sevoflurane-induced cognitive impairment in aged mice through brain-derived neurotrophic factor/tyrosine receptor kinase B and neurotrophin-3/tropomyosin receptor kinase C pathways. Front Aging Neurosci.

[38] Gonçalves JT, Schafer ST, Gage FH (2016). Adult neurogenesis in the hippocampus: from stem cells to behavior. Cell.

[39] Hovens IB, van Leeuwen BL, Mariani MA, Kraneveld AD, Schoemaker RG (2016). Postoperative cognitive dysfunction and neuroinflammation; cardiac surgery and abdominal surgery are not the same. Brain Behav Immun.

[40] Lang HL, Zhao YZ, Xiao RJ, Sun J, Chen Y, Hu GW, Xu GH (2023). Small extracellular vesicles secreted by induced pluripotent stem cell-derived mesenchymal stem cells improve postoperative cognitive dysfunction in mice with diabetes. Neural Regen Res.

[41] Fan D, Li J, Zheng B, Hua L, Zuo Z (2016). Enriched environment attenuates surgery-induced impairment of learning, memory, and neurogenesis possibly by preserving BDNF expression. Mol Neurobiol.

[42] Deshpande A, Bergami M, Ghanem A, Conzelmann KK, Lepier A, Götz M, Berninger B (2013). Retrograde monosynaptic tracing reveals the temporal evolution of inputs onto new neurons in the adult dentate gyrus and olfactory bulb. Proc Natl Acad Sci U S A.

[43] Vivar C, Potter MC, Choi J, Lee JY, Stringer TP, Callaway EM, Gage FH, Suh H, van Praag H (2012). Monosynaptic inputs to new neurons in the dentate gyrus. Nat Commun.

[44] Ge S, Goh EL, Sailor KA, Kitabatake Y, Ming GL, Song H (2006). GABA regulates synaptic integration of newly generated neurons in the adult brain. Nature.

[45] Zhao C, Teng EM, Summers RG, Ming GL, Gage FH (2006). Distinct morphological stages of dentate granule neuron maturation in the adult mouse hippocampus. J Neurosci.

[46] Shors TJ, Miesegaes G, Beylin A, Zhao M, Rydel T, Gould E (2001). Neurogenesis in the adult is involved in the formation of trace memories. Nature.

[47] van Praag H, Schinder AF, Christie BR, Toni N, Palmer TD, Gage FH (2002). Functional neurogenesis in the adult hippocampus. Nature.

[48] Unger MS, Li E, Scharnagl L, Poupardin R, Altendorfer B, Mrowetz H, Hutter-Paier B, Weiger TM, Heneka MT, Attems J, Aigner L (2020). CD8(+) T-cells infiltrate Alzheimer’s disease brains and regulate neuronal- and synapse-related gene expression in APP-PS1 transgenic mice. Brain Behav Immun.

[49] Louveau A, Herz J, Alme MN, Salvador AF, Dong MQ, Viar KE, Herod SG, Knopp J, Setliff JC, Lupi AL (2018). CNS lymphatic drainage and neuroinflammation are regulated by meningeal lymphatic vasculature. Nat Neurosci.

[50] Carrasco E, Gómez de Las Heras MM, Gabandé-Rodríguez E, Desdín-Micó G, Aranda JF, Mittelbrunn M (2022). The role of T cells in age-related diseases. Nat Rev Immunol.

[51] Goddery EN, Fain CE, Lipovsky CG, Ayasoufi K, Yokanovich LT, Malo CS, Khadka RH, Tritz ZP, Jin F, Hansen MJ, Johnson AJ (2021). Microglia and perivascular macrophages act as antigen presenting cells to promote CD8 T cell infiltration of the brain. Front Immunol.

[52] Wu J, Guo Y, Li W, Zhang Z, Li X, Zhang Q, Du Q, Niu X, Liu X, Wang G (2023). Microglial priming induced by loss of Mef2C contributes to postoperative cognitive dysfunction in aged mice. Exp Neurol.

[53] Ritzel RM, Crapser J, Patel AR, Verma R, Grenier JM, Chauhan A, Jellison ER, McCullough LD (2016). Age-associated resident memory CD8 T cells in the central nervous system are primed to potentiate inflammation after ischemic brain injury. J Immunol.

[54] Garber C, Soung A, Vollmer LL, Kanmogne M, Last A, Brown J, Klein RS (2019). T cells promote microglia-mediated synaptic elimination and cognitive dysfunction during recovery from neuropathogenic flaviviruses. Nat Neurosci.

[55] Shih L, Guler N, Syed D, Hopkinson W, McComas KN, Walborn A, Hoppensteadt D, Fareed J, Rondina MT (2018). Postoperative changes in the systemic inflammatory milieu in older surgical patients. Clin Appl Thromb Hemost.

[56] Sun Z, Mao A, Wang Y, Zhao Y, Chen J, Xu P, Miao C (2017). Treatment with anti-programmed cell death 1 (PD-1) antibody restored postoperative CD8+ T cell dysfunction by surgical stress. Biomed Pharmacother.

[57] Monteiro S, Ferreira FM, Pinto V, Roque S, Morais M, de Sá-Calçada D, Mota C, Correia-Neves M, Cerqueira JJ (2016). Absence of IFNγ promotes hippocampal plasticity and enhances cognitive performance. Transl Psychiatry.

[58] Ta TT, Dikmen HO, Schilling S, Chausse B, Lewen A, Hollnagel JO, Kann O (2019). Priming of microglia with IFN-γ slows neuronal gamma oscillations in situ. Proc Natl Acad Sci U S A.

[59] Borsini A, Zunszain PA, Thuret S, Pariante CM (2015). The role of inflammatory cytokines as key modulators of neurogenesis. Trends Neurosci.

[60] Zhang J, He H, Qiao Y, Zhou T, He H, Yi S, Zhang L, Mo L, Li Y, Jiang W, You Z (2020). Priming of microglia with IFN-γ impairs adult hippocampal neurogenesis and leads to depression-like behaviors and cognitive defects. Glia.

