# Supplementary figures and images for: Inhibition of adult hippocampal neurogenesis induced by postoperative CD8 + T-cell infiltration is associated with cognitive decline later following surgery in adult mice
Source: J Neuroinflammation. 2023 Oct 5;20:227. doi: 10.1186/s12974-023-02910-x (PMC10557222; doi:10.1186/s12974-023-02910-x)

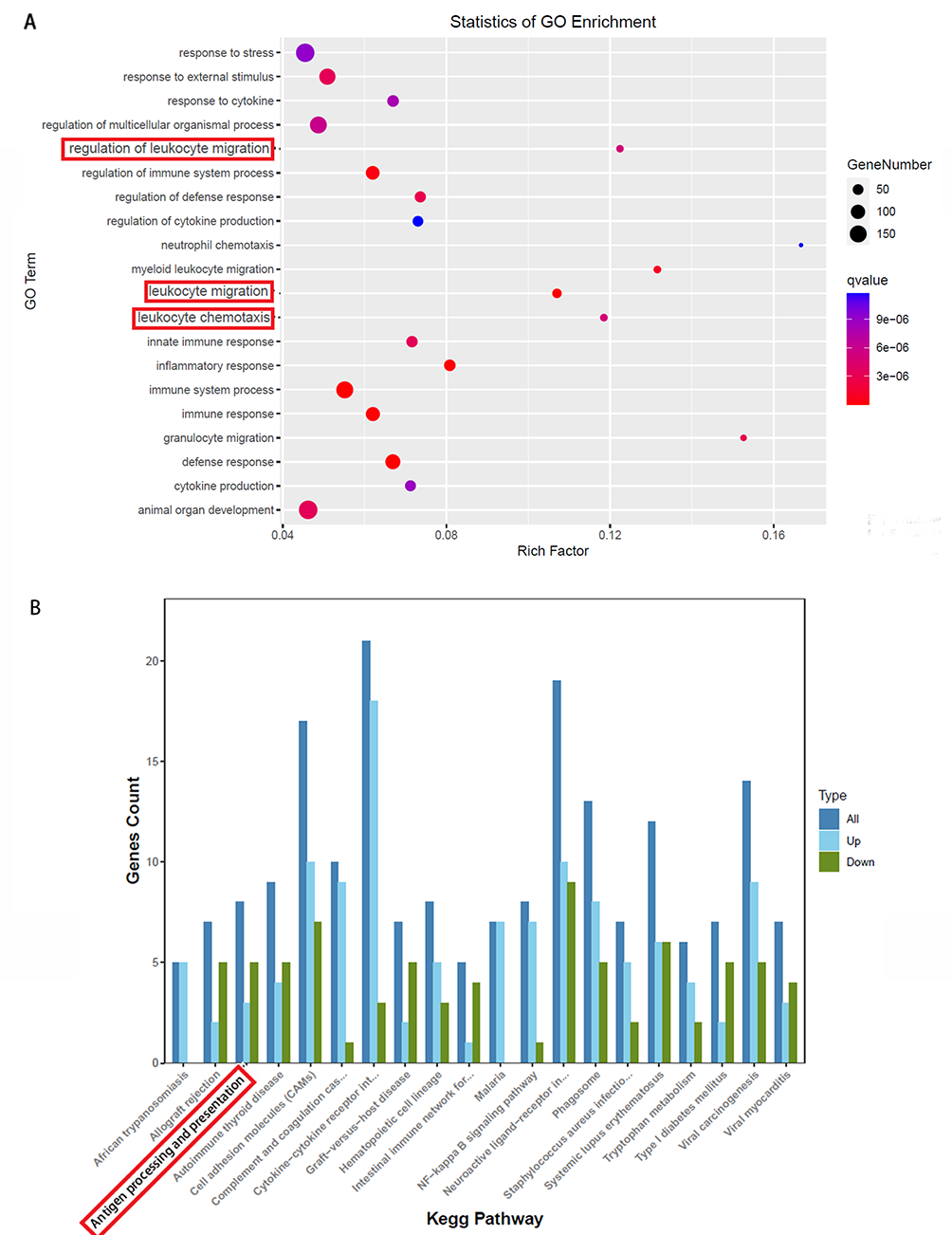

Supplement: Supplementary file 1 — Additional file 1: Figure S1. RNA sequencing of the hippocampus suggested the possibility of leukocyte infiltration after surgery. (A) GO enrichment analysis; (B) KEGG pathway analysis. [file 12974_2023_2910_MOESM1_ESM.tif]

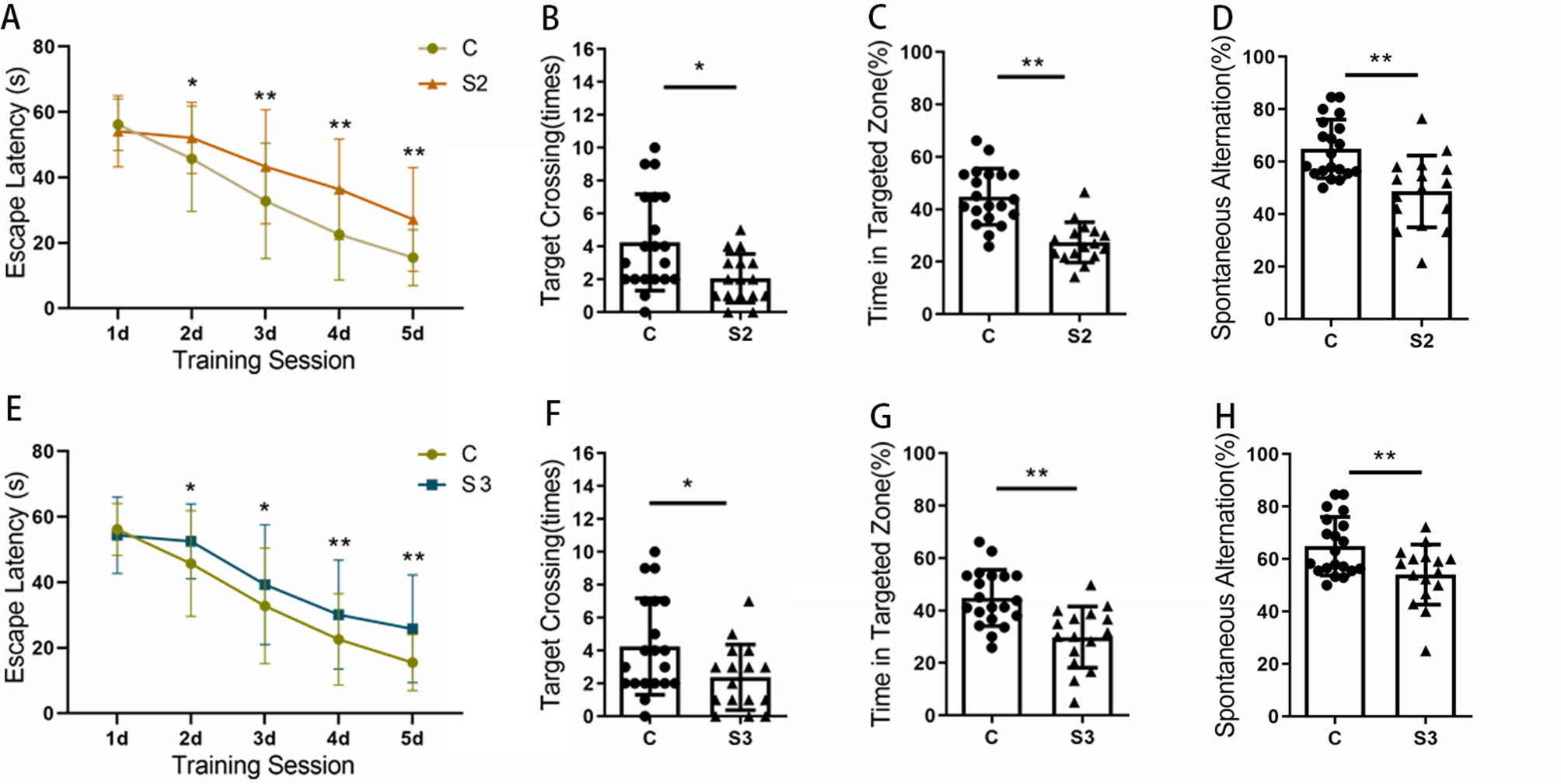

Supplement: Supplementary file 2 — Additional file 2: Figure S2. Learning and memory performance at week 2 and week 3 after surgery. (A), (E) Escape latencies during training days; (B), (F) Times of target crossings on the testing day; (C), (G) Percentages of time spent in the targeted zone on the testing day; (D), (H) Spontaneous alternation rate in the Y maze. C, control; S2/S3, animals that underwent behavioral testing during weeks 2/3 after surgery. N = 16–20/group. * p < 0.05, ** p < 0.01. [file 12974_2023_2910_MOESM2_ESM.tif]

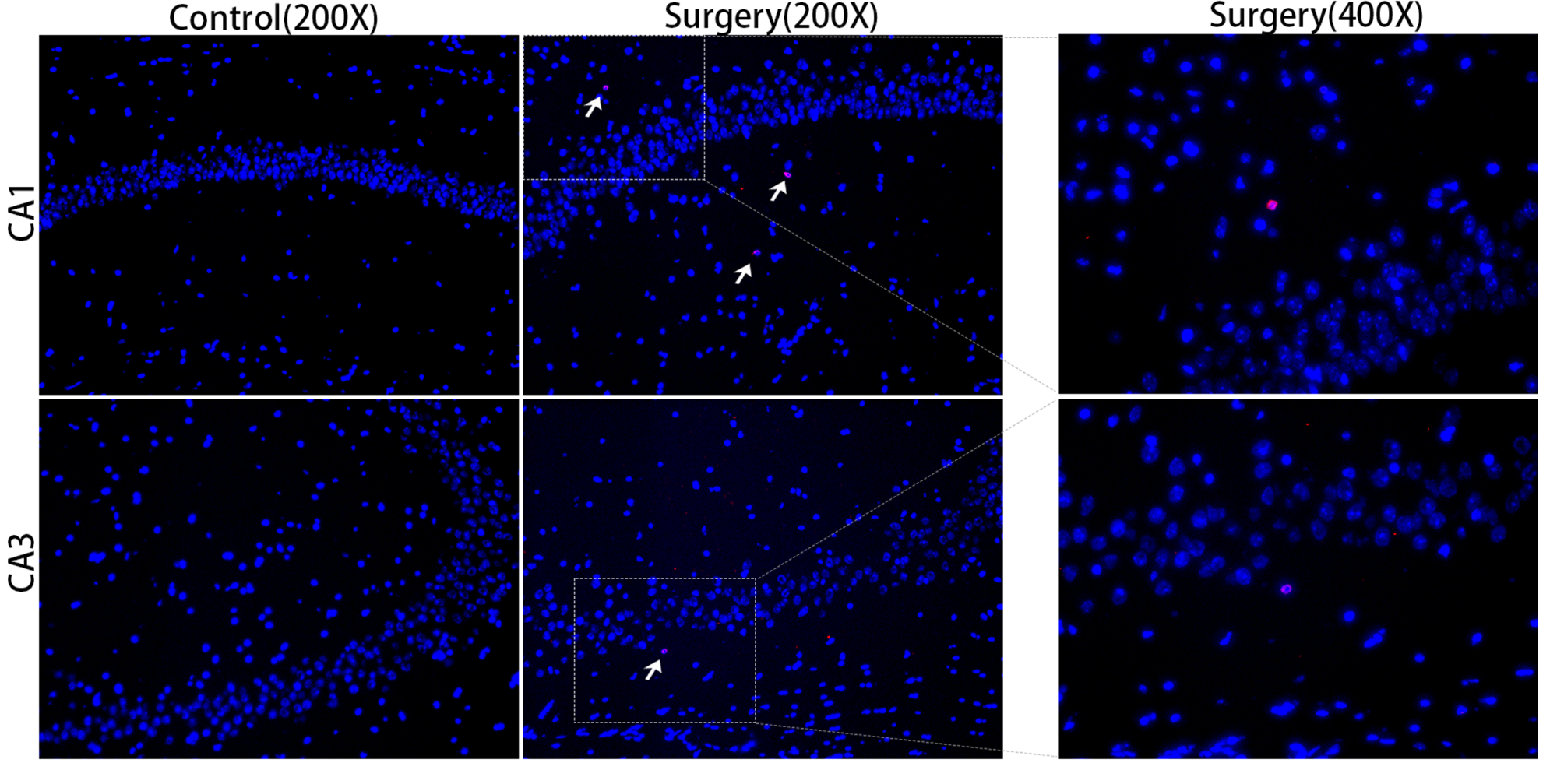

Supplement: Supplementary file 3 — Additional file 3: Figure S3. Representative immunofluorescence image of CD3 + T cells in the hippocampus of mice after surgery. [file 12974_2023_2910_MOESM3_ESM.tif]
